# A Programmable, 3D Neuron‐On‐Chip Platform Integrating Near Real‐Time Biosensing and Multiaxial Loading for Mechanobiological Injury Profiling

**DOI:** 10.1002/advs.202510309

**Published:** 2025-10-07

**Authors:** Sultan Khetani, Kar Wey Yong, Mawafag F. Alhasadi, Amir Hamedzadeh, Leila Shahsavari, Atefeh Rafiei, Kalvin Wu, Omakhowa Agbojo, Anupriya Singh, Kunal Karan, Salvatore Federico, Arindom Sen, Amir Sanati‐Nezhad

**Affiliations:** ^1^ BioMEMS and Bioinspired Microfluidic Laboratory Department of Biomedical Engineering University of Calgary Calgary Alberta T2N 1N4 Canada; ^2^ Graduate Program in Biomedical Engineering University of Calgary Calgary Alberta T2N 1N4 Canada; ^3^ Pharmaceutical Production Research Facility Schulich School of Engineering University of Calgary Alberta T2N 1N4 Canada; ^4^ Department of Mechanical and Manufacturing Engineering Schulich School of Engineering University of Calgary Calgary Alberta T2N 1N4 Canada; ^5^ Department of Chemical and Petroleum Engineering University of Calgary Calgary Alberta T2N 1N4 Canada; ^6^ Department of Biomedical Engineering Schulich School of Engineering University of Calgary Calgary Alberta T2N 1N4 Canada

**Keywords:** central nervous system injury, electrochemical biosensors, mechanical loading, neuron filament light protein, neuron‐injury‐on‐a‐chip, total‐tau protein

## Abstract

Mechanical forces imparted to the central nervous system (CNS) generate complex, load‐dependent injury patterns, yet the molecular mechanisms linking physical insult to biomarker response remain poorly defined. Here, the Neuron‐Injury‐on‐a‐Chip (NIOC) platform is presented as a programmable 3D microfluidic system integrating multiaxial loading with real‐time biosensing. The system features a polydimethylsiloxane (PDMS) tube internally coated with polydopamine to support Cath. a‐differentiated (CAD) neuron adhesion and viability. Controlled extension, torsion, and combined loads simulate physiologically relevant CNS trauma. Finite element modeling confirms uniform strain transmission, while embedded electrochemical biosensors enable near real‐time detection of total tau (T‐Tau) and neurofilament light chain (NFL) at picogram levels. qPCR and immunostaining validate gene‐level responses (Mapt, Gap‐43) and apoptosis (Caspase‐3). Load‐specific biomarker trajectories and apoptotic thresholds are uncovered, with synergistic injury responses under combined loading. NIOC represents a first‐in‐class platform for decoding mechanobiological injury, offering new opportunities for biomarker discovery, injury stratification, and neuroprotective screening.

## Graphical abstract

A programmable 3D Neuron‐Injury‐on‐a‐Chip platform integrates multiaxial mechanical loading with ultrasensitive electrochemical biosensing to decode neuronal responses in real time. By mimicking physiologically relevant extension–torsion forces, the system reveals load‐specific biomarker trajectories (T‐Tau, NFL), gene regulation, and apoptosis. This approach enables mechanobiological injury profiling, advancing biomarker discovery, therapeutic screening, and neurotrauma diagnostics.

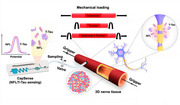

## 1 Introduction

The central nervous system (CNS)—comprising the brain and spinal cord—is highly sensitive to mechanical perturbations that can produce traumatic brain injury (TBI), spinal cord injury (SCI), concussion, or subconcussive insult.^[^ ^1^, ^2^ ^]^ Each year, more than 90 million people worldwide sustain CNS injuries,^[^ ^3^ ^]^ initiating complex cascades of structural and molecular damage such as contusion, vascular compromise, axonal shearing, and neuroinflammation.^[^ ^4^ ^]^ Clinically, TBI is categorized as mild, moderate, or severe based on symptoms and radiologic findings;^[^ ^5^ ^]^ however, this framework overlooks molecular heterogeneity and frequently misses subclinical or evolving pathologies—especially in mild cases where imaging is normal but symptoms persist. These gaps have driven demand for objective, biologically grounded tools to detect injury early and discriminate among loading modes and severities.

Beyond neuroimaging, fluid‐phase protein biomarkers provide a minimally invasive, temporally responsive measure of neuronal integrity, particularly during the acute phase.^[^ ^6^ ^]^ Total tau (T‐Tau) and neurofilament light chain (NFL) are rapidly released into cerebrospinal fluid (CSF) and blood after axonal damage.^[^ ^7^ ^]^ Elevated T‐Tau and NFL levels stratify injury severity, inform return‐to‐play decisions, and predict long‐term neurodegeneration.^[^ ^8^ ^]^ Advances in biosensing—especially electrochemical immunosensors—now enable high‐sensitivity detection of these markers at or near the point of care ^[^ ^9^ ^]^.

Recent reviews compile clinical ranges and assay performance for T‐Tau, NFL, GFAP, and UCH‐L1 and stress the need for early, serial sampling and platforms that can resolve load‐specific biomarker kinetics.^[^ ^10^, ^11^ ^]^ Yet capturing the precise, load‐dependent release dynamics remains difficult because serial biofluid sampling within minutes to hours of injury is impractical or ethically constrained in patients and animal models. This temporal gap limits our understanding of how discrete mechanical forces—extension, torsion, or combined multiaxial stress—map onto early biomarker signatures when therapeutic intervention is most effective ^[^ ^12^ ^]^.

At cellular and subcellular scales, mechanical insults disrupt neuronal homeostasis and axonal integrity. Acute axonal deformation causes mechanoporation of membranes, ionic imbalance, calcium influx, and cytoskeletal disruption.^[^ ^13^, ^14^ ^]^ These primary events trigger mitochondrial dysfunction, oxidative stress, and caspase activation, culminating in apoptosis and progressive axonal degeneration.^[^ ^15^, ^16^ ^]^ Diffuse axonal injury (DAI)—a hallmark of TBI—is characterized by widespread disconnection and long‐term neurological deficits ^[^ ^17^ ^]^ and may occur even without visible lesions on neuroimaging.^[^ ^18^ ^]^ Subclinical or repetitive insults can also induce persistent microstructural damage, glial activation, and pathological protein accumulation, increasing risk for chronic traumatic encephalopathy (CTE), Alzheimer's disease, and related neurodegenerative conditions.^[^ ^19^ ^]^ These findings underscore the need for physiologically relevant platforms that replicate CNS mechanical microenvironments and reveal the molecular consequences of specific loading modalities.

Mechanical loading is a critical determinant of neuronal injury outcomes, yet the distinct effects of uniaxial stretch, torsion, and multiaxial combinations remain under‐characterized at the cellular and molecular levels. In vivo, brain tissue often experiences irregular, multiaxial stresses from blunt impacts, rapid acceleration–deceleration, or blast waves, producing heterogeneous patterns of axonal deformation and shearing.^[^ ^20^ ^]^ The type and geometry of force dictate where strain localizes, how microtubules rupture, and how cytoskeletal and membrane structures reorganize.^[^ ^21^ ^]^ Torsional shear, for example, is strongly implicated in diffuse axonal injury, whereas tensile strain can disrupt axonal polarity and elongation.^[^ ^22^ ^]^ Although biomechanical models predict deformation thresholds for different loading modes,^[^ ^23^, ^24^ ^]^ experimental evidence linking these forces to acute molecular events—protein release, transcriptional changes, and apoptosis—remains limited due to challenges in capturing high‐temporal‐resolution biosignatures.^[^ ^25^, ^26^ ^]^ Addressing this gap requires platforms capable of applying programmable, physiologically relevant forces while simultaneously monitoring neuronal responses in situ.

Conventional in vitro systems—including 2D stretch chambers, compression devices, and shear‐flow bioreactors—have illuminated basic cell responses to mechanical stress.^[^ ^27^, ^28^ ^]^ Yet, their planar cultures lack the 3D architecture, matrix stiffness, and complex cell–matrix interactions of native CNS tissue.^[^ ^29^, ^30^ ^]^ Moreover, most rely on post hoc assays such as ELISA, western blotting, or fixed‐cell staining.^[^ ^33^, ^34^ ^]^ which require pooling of samples, consume large reagent volumes, and lack temporal resolution for dynamic or longitudinal analyses. Spheroids and hydrogel‐based neuronal constructs partially address spatial organization and mechanical context ^[^ ^31^, ^32^ ^]^ but rarely include integrated sensing or programmable loading.^[^ ^33^, ^34^ ^]^ Recent organ‐on‐a‐chip innovations overcome many of these shortcomings by combining engineered 3D scaffolds, tunable mechanical actuation, and perfusable microfluidic architectures.^[^ ^35^, ^36^ ^]^ Reviews highlight rapid advances in brain‐on‐chip platforms—including sensor‐integrated systems and disease models—and their value for mechanobiology and neurotrauma research.^[^ ^37^, ^38^, ^39^ ^]^ Compared to animal models, these miniaturized systems provide superior spatiotemporal control, reproducibility, and ethical advantages, while enabling high‐throughput, multiplexed experiments with near real‐time analytical readouts ^[^ ^40^, ^41^ ^]^.

**Scheme 1:**
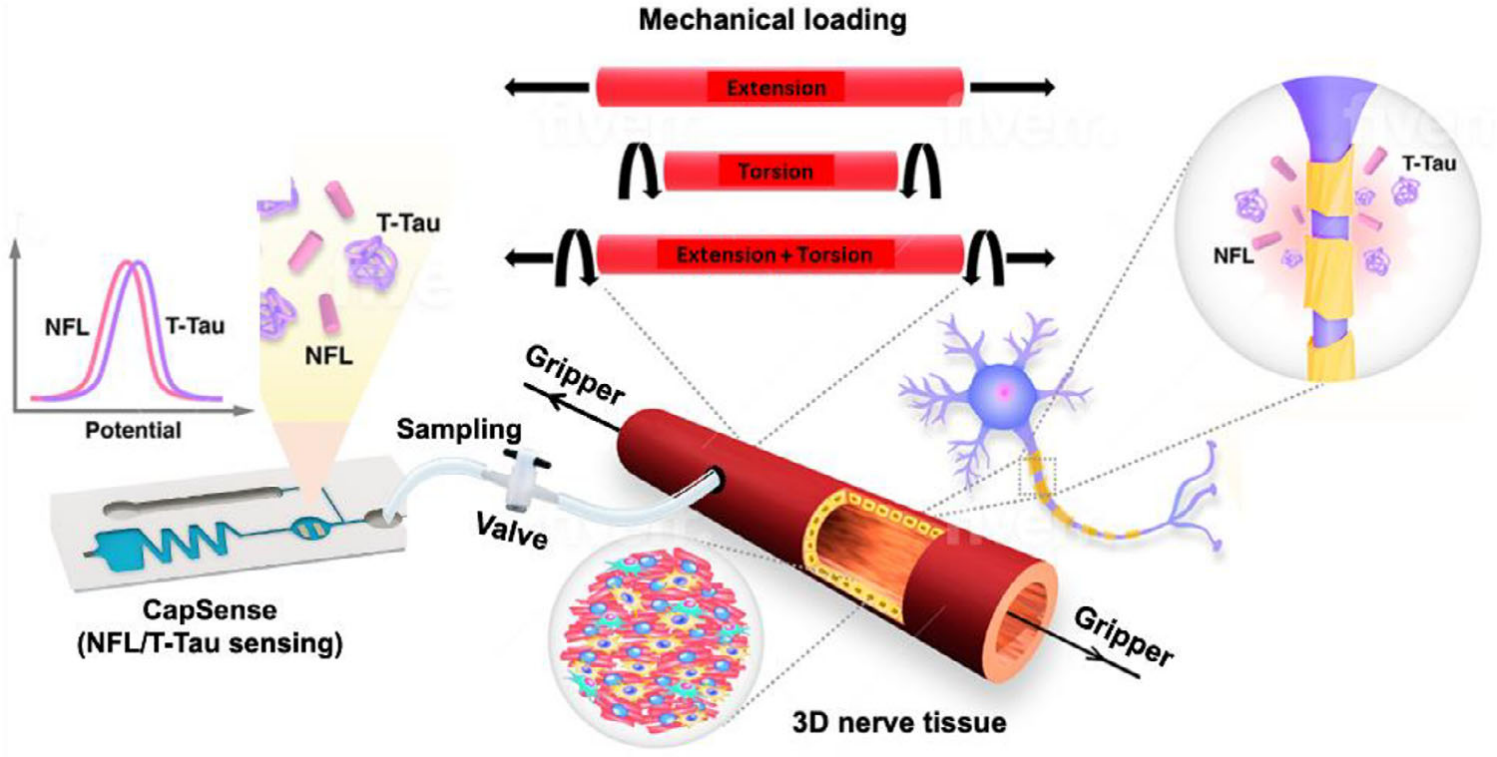
Design and function of the Neuron‐Injury‐on‐a‐Chip (NIOC) platform integrating programmable mechanical loading and near real‐time biosensing. The cylindrical microfluidic chip, fabricated from polydimethylsiloxane (PDMS) and seeded with Cath.a‐differentiated (CAD) neurons, is subjected to tunable extension, torsion, or combined multiaxial loading to mimic biomechanical injury conditions. Mechanical deformation induces neuronal injury and triggers the release of protein biomarkers—total tau (T‐Tau) and neurofilament light chain (NFL)—into the surrounding microenvironment. Integrated electrochemical biosensors, embedded within the NIOC architecture, enable label‐free, near real‐time quantification of these biomarkers at picogram sensitivity. The system provides spatiotemporally resolved insight into load‐dependent injury signatures, establishing a foundation for mechanobiological injury profiling in 3D neural tissues. PDMS tube connected downstream via narrow tubing to a valve and a CapSense dual electrochemical sensing chip.^[^ ^42^ ^]^ This arrangement enables automated sampling during mechanical loading without disrupting device function or impacting the cell culture environment.

To address the need for physiologically relevant, real‐time monitoring of mechanobiological injury pathways, we developed a programmable, 3D Neuron‐Injury‐on‐a‐Chip (NIOC) platform that integrates multiaxial loading with on‐chip biosensing capabilities (**Scheme** **1**). The system consists of a cylindrical polydimethylsiloxane (PDMS) microtube seeded with Cath. a‐differentiated (CAD) neurons, coated internally with polydopamine to support cellular adhesion and uniform distribution. This architecture enables the robust application of precisely controlled extension (4%–20%, ∆4%), torsion (30°–300°, ∆30°), and combined extension–torsion (4%–12%, ∆4%; 30°–90°, ∆30°) to mimic the heterogeneous loading conditions characteristic of CNS trauma. Critically, our platform incorporates multiplexed electrochemical biosensors with the NIOC microfluidic chip, allowing near real‐time, label‐free detection of injury‐related biomarkers—including total tau (T‐Tau) and neurofilament light chain (NFL)—with picogram‐level sensitivity and minimal sample volume. These sensors offer temporal granularity suitable for capturing acute injury kinetics. Their integration within the NIOC system eliminates the need for bulk sample pooling and post hoc analysis, allowing longitudinal tracking of biomarker release in situ. Coupling this sensing capability with transcriptional analysis of key mechanosensitive genes (e.g., Mapt, Gap‐43) allows us to correlate mechanical input with proteomic release and genomic activation, providing a systems‐level view of neuronal injury. The system allows precise mapping of injury thresholds by capturing the dynamic secretion profiles of clinically relevant proteins following extension, torsion, and combined mechanical insults. It facilitates mechanistic analysis of biomarker trajectories in response to physical stress.

## 2 Results and Discussion

### 2.1 Engineering of Neuron‐Injury‐On‐a‐Chip (NIOC) Platform

**Figure 1:**
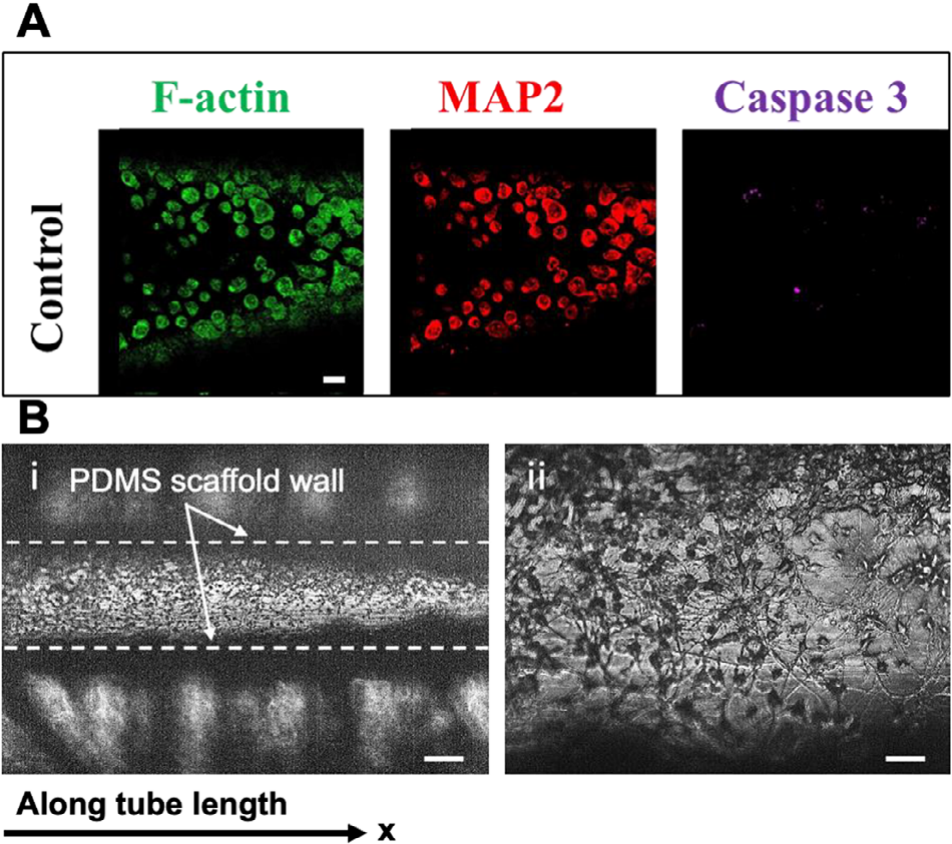
Establishment of 3D neuronal culture within the cylindrical NIOC platform. A) Immunofluorescence micrographs of CAD neurons cultured for 5 days inside polydopamine‐ and collagen‐coated PDMS microtubes, stained for F‐actin (green), MAP2 (red), and cleaved Caspase‐3 (purple). The dashed lines indicate the approximate channel boundaries along the tube length. The dense central band corresponds to the CAD neuronal layer adhered to the PDMS inner wall. Fine radial projections represent extending axons and dendrites oriented along the curvature of the channel. The outer gray boundary represents the PDMS scaffold wall. Extensive F‐actin and MAP2 expression confirm robust cytoskeletal organization and neuronal differentiation, while minimal Caspase‐3 signal (<5%) indicates low baseline apoptosis. Scale bars: 200 µm. B) Bright‐field images of the same 3D neuronal culture: i) longitudinal view of the cylindrical tube (dashed lines outline the channel boundaries) showing the neuronal layer and interior projections, and ii) higher‐magnification view highlighting dense neurite networks extending along and around the tube interior. Axonal projections exhibit random orientation, indicating spontaneous neurite outgrowth in the absence of imposed alignment cues. Scale bars: 5 mm i), 500 µm ii).

The cylindrical 3D polymeric microfluidic chip provided a physiologically relevant scaffold for reconstructing in vitro neuronal tissue and assessing responses to mechanical stress. Detailed chip fabrication methods are presented in the Experimental Section. Designed to mimic key features of in vivo neuronal injury, the chip supports 3D cellular organization and enables near real‐time monitoring of mechanically induced neurodegeneration. Although CAD cells are of murine origin, they exhibit neuronal phenotypes and are widely used for the central nervous system (CNS) modeling.^[^ ^43^, ^44^ ^]^ For the development of the Neuron‐Injury‐On‐a‐Chip (NIOC) platform, CAD cells were seeded in polydopamine (PDA)‐ and collagen‐coated microchannels (**Figure** **1**) and cultured for four days in serum‐enriched medium, followed by one day in serum‐free medium to promote axonal extension ^[^ ^43^ ^]^.

Bright‐field and immunofluorescence imaging confirmed the successful establishment of a viable and spatially organized 3D neuronal culture within the cylindrical PDMS microfluidic tube. As shown in Figure 1A, CAD cells cultured for 5 days (3 days in serum‐containing medium followed by 2 days in serum‐free medium) exhibited robust attachment and differentiation, evidenced by strong MAP2 expression (red), indicating dendritic maturation and F‐actin staining (green), confirming cytoskeletal organization and spreading along the inner tube wall. Minimal cleaved Caspase‐3 signal (purple) was observed, with apoptotic cells accounting for less than 5% of the population, suggesting low baseline cytotoxicity and high viability under static conditions. Figure 1B displays bright‐field images from the same culture period, highlighting dense neuronal networks and extensive neurite outgrowth. Numerous axonal projections can be seen extending both radially and longitudinally along the tubular architecture, with random orientation and no imposed alignment, consistent with spontaneous axonal growth in a 3D environment. The visible presence of axons growing in diverse directions underscores the permissive and supportive nature of the microenvironment. Together, these data validate the effectiveness of the PDMS‐PDA‐collagen composite scaffold in supporting neuronal adhesion, viability, and neurite extension, and establish a physiologically relevant baseline for subsequent biomechanical injury modeling within the NIOC platform.

### 2.2 Surface Stability validation Via Mass Spectrometry

To confirm the chemical stability of the Tube's PDA coating under mechanical stress, we performed matrix‐assisted laser desorption/ionization time‐of‐flight mass spectrometry (MALDI‐TOF MS) on culture medium sampled 2 h post‐loading. PDA serves as the primary adhesion layer for neuronal culture in the NIOC system, yet its durability under torsion and extension forces has not been previously validated in dynamic systems. No characteristic peaks associated with dopamine monomers, PDA oligomers, or other degradation products were detected in conditioned media, confirming that the coating remained intact throughout the loading cycles. These findings eliminate the possibility of mechanical delamination or biochemical contamination from PDA, reinforcing the suitability of this surface chemistry for long‐term, real‐time biosensing in mechanically active environments (see Table S2 and; Figure S7, Supporting Information). The chemical and mechanical stability of PDA coatings on PDMS under dynamic culture conditions has been extensively validated in prior work, including our own. In earlier studies,^[^ ^43^, ^44^ ^]^ PDA‐functionalized PDMS microchannels maintained neuronal and tissue cultures for at least four weeks under continuous perfusion without detectable leaching or loss of adhesion. Similar long‐term durability has been reported in a range of biomedical contexts, including >8 weeks of underwater adhesion stability ^[^ ^45^ ^]^ and >12 weeks on in vivo implants.^[^ ^46^ ^]^ These findings, together with the MALDI‐TOF confirmation of no PDA degradation after repeated extension/torsion cycles in the present study, indicate that PDA coatings on PDMS are suitable for extended use and can be directly applied in future chronic neurotrauma monitoring models.

### 2.3 Programmable Mechanical Loading to Simulate Biomechanical Trauma

In real‐life scenarios such as sports‐related impacts, vehicular accidents, falls, or blast exposures, the brain is subjected not only to linear acceleration forces (e.g., direct translational impact) but also to complex biomechanical loading, including stretching (extension), rotational shearing (torsion), and combined multiaxial stresses. These forces lead to rapid deformation of neural tissues, particularly axons, and are key contributors to DAI—a defining pathology in concussion and TBI.^[^ ^47^, ^48^ ^]^ Among these forces, rotational or torsional loads are especially implicated in axonal shearing and are considered more damaging than linear forces alone.^[^ ^49^ ^]^ Extension or uniaxial stretch, on the other hand, affects membrane permeability, cytoskeletal dynamics, and synaptic signaling.^[^ ^50^ ^]^ Importantly, combined extension and torsion closely mimic the heterogeneous deformations encountered during real‐world traumatic events, where tissues often undergo simultaneous stretching and twisting, as seen in football tackles, falls with head rotation, rear‐end collisions (whiplash), and blast injuries ^[^ ^51^ ^]^.

**Figure 2:**
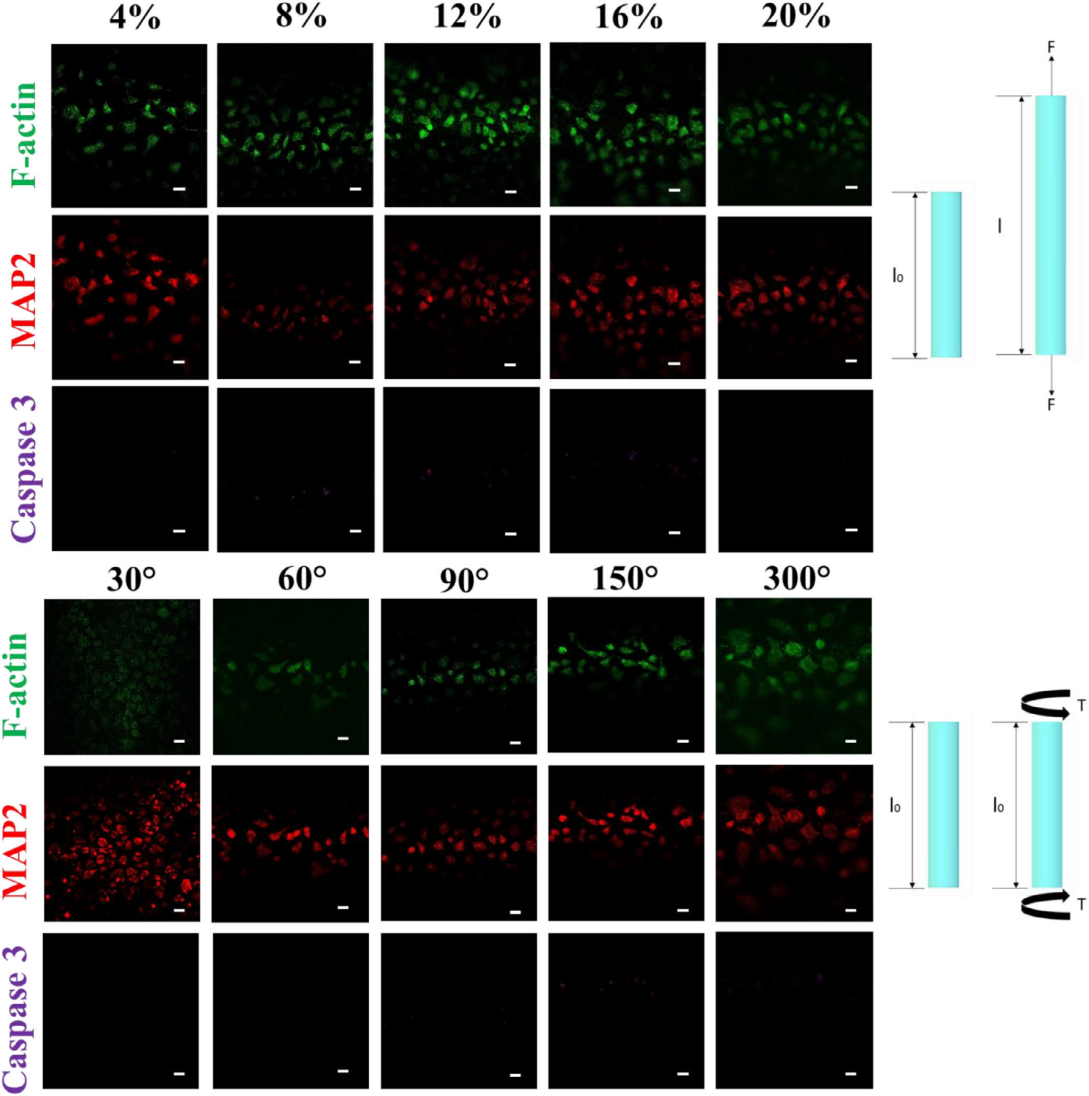
Response to uniaxial extension and torsion. Immunofluorescent images of CAD cells following mechanical loading by uniaxial extension and torsion. MAP2 (red) staining confirmed the preservation of neuronal phenotype, while cleaved Caspase‐3 (purple) staining showed no significant increase in apoptotic cells. CAD cells were exposed to increasing levels of extension (4–20%) and torsion (30°–300°). Scale bar: 200 µm.

Despite their physiological relevance, existing in vitro platforms seldom provide well‐defined, quantifiable loading regimens—let alone the capability for near–real‐time monitoring of neuronal responses.^[^ ^41^ ^]^ To address these limitations, we used the 3D Neuron‐Injury‐on‐a‐Chip (NIOC) system to deliver precisely controlled axial extension (4–20%), torsion (30°–300°), and combined extension–torsion loads (4–12% + 30°–90°) to CAD neuronal cultures (**Figure** **2**). These programmed conditions deliberately span sub‐concussive, near‐threshold, and suprathreshold regimes, enabling systematic stratification of cellular responses by load geometry and intensity. The chosen strain and twist values correspond to deformation magnitudes (maximum principal strains of ≈0.10–0.25) that have been consistently associated with axonal injury in experimental stretch studies and validated finite‐element head models of concussive and sub‐concussive TBI ^[^ ^27^, ^52^, ^53^, ^54^ ^]^.

Our results reveal that neuronal tissue exposed to isolated uniaxial extension or torsion exhibits strong structural resilience: MAP2 expression remained stable and Caspase‐3 activation was minimal, suggesting preservation of neuronal identity and low apoptosis. These responses closely mirror subclinical mild traumatic brain injury (mTBI), where diffuse axonal stress may not produce overt structural lesions yet initiate subtle neurobiological perturbations.^[^ ^55^ ^]^ Clinically, these findings are significant. In contact sports such as football, soccer, and hockey, repetitive subthreshold head impacts can cumulatively impair neural integrity despite an absence of acute symptoms.^[^ ^56^ ^]^ Likewise, whiplash injuries from vehicular collisions often involve complex rotational and tensile loads that rarely yield radiologically detectable damage yet contribute to persistent symptoms such as cognitive dysfunction, dizziness, and headache.^[^ ^57^ ^]^ The inability of current diagnostic tools to detect these low‐grade injuries underscores the need for high‐resolution, mechanistically informed platforms like NIOC that can delineate injury thresholds with cellular precision.

**Figure 3:**
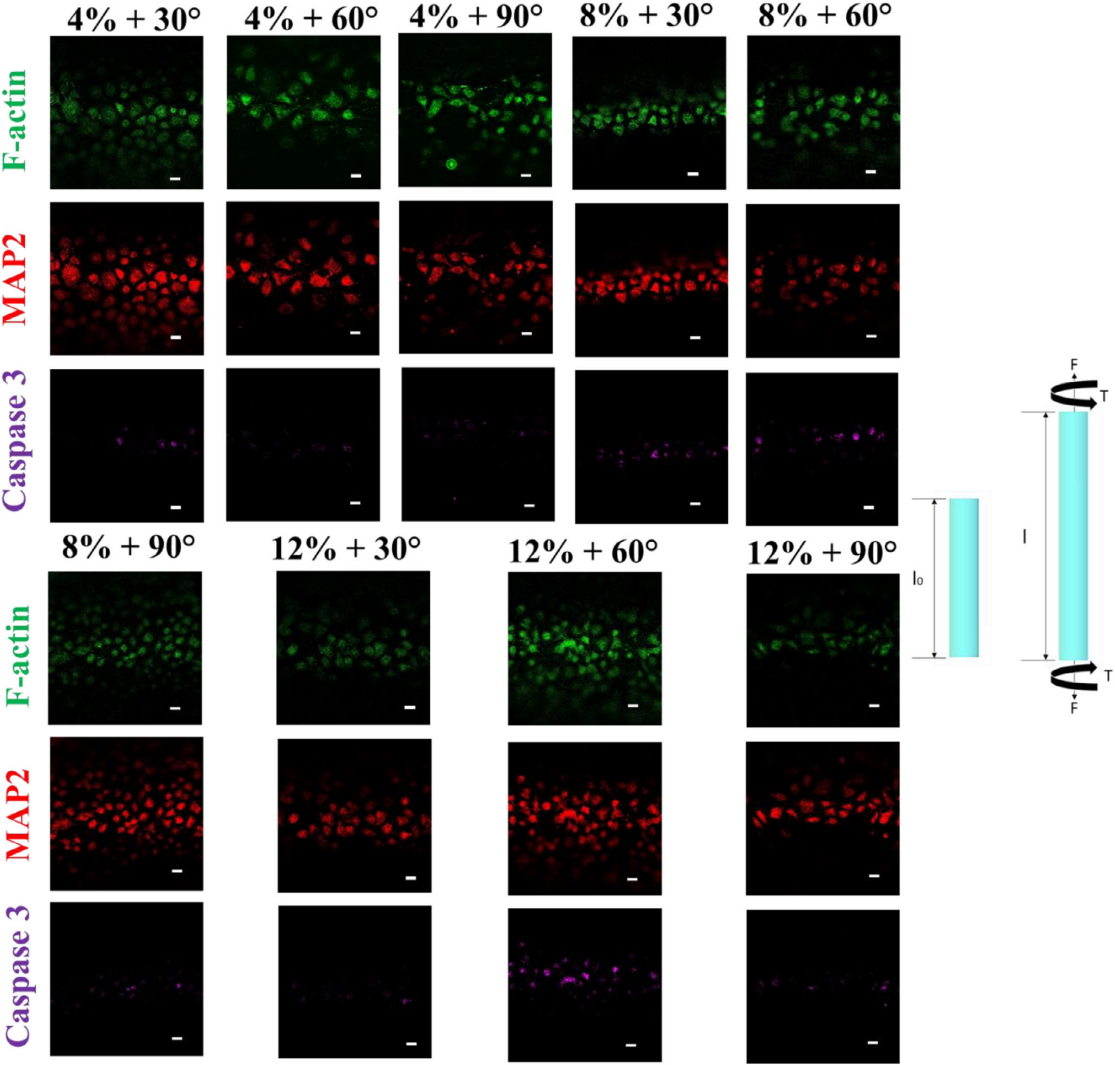
Fluorescent images of CAD cells exposed to combined mechanical loading (extension + torsion). MAP2 expression (red) was sustained, but Caspase‐3+ staining (purple) increased significantly in higher combined loading conditions, indicating activation of apoptotic pathways. Scale bar: 200 µm.

The application of combined mechanical loads—specifically 8–12% extension with 60°–90° torsion—led to a pronounced increase in neuronal apoptosis, with Caspase‐3+ cells rising from a baseline of ∼5% in static conditions to 20–25% (**Figure** **3**). This finding confirms the high sensitivity of neurons to multiaxial loading, beyond the isolated effects of either extension or torsion alone. Notably, MAP2 expression remained largely unchanged across these conditions, indicating that while a subpopulation of neurons underwent apoptotic signaling, others maintained structural integrity and neuronal identity.^[^ ^26^ ^]^ Data are consistent with neuropathological findings from human TBI and animal models, where rotational and shear strains—common in whiplash injuries, contact sports, and blast exposure—are primary contributors to DAI.^[^ ^49^ ^]^ Axonal shearing caused by torsional or combined loading is known to initiate cytoskeletal breakdown and apoptotic cascades. Our results align with such pathophysiological observations: apoptosis increased sharply with combined loading, but the persistence of MAP2+ cells suggests that injury was spatially or temporally heterogeneous, as also reported in histological analyses of moderate TBI cases. The non‐linear increase in Caspase‐3 activation at higher combined loads also supports the presence of a mechanical threshold beyond which neuronal viability declines sharply. Similar threshold behavior has been reported in brain tissue subjected to angular acceleration or high‐rate deformation, where exceeding critical strain magnitudes results in irreversible injury.^[^ ^52^ ^]^ The fact that Caspase‐3 activation was minimal under isolated loading but significantly elevated under combined stress reinforces the role of loading geometry in injury severity. The results provide a quantitative framework for linking specific mechanical load parameters to neuronal injury mechanisms observed in vivo. They reinforce findings that combined deformation—rather than force magnitude alone—plays a decisive role in triggering apoptosis. These data also support the distinction between structural preservation (MAP2) and functional viability (Caspase‐3 activation), highlighting the complexity of neuronal injury progression even under controlled mechanical conditions.

### 2.4 Molecular Profiling Reveals Load‐Specific Neurodegenerative Signatures

To further investigate the mechanistic effects of defined biomechanical insults, we quantified changes in the expression of key TBI and neuronal markers at the gene level and assessed apoptosis using immunostaining. Specifically, we measured the mRNA expression of *Mapt* (encoding Tau), *Gap‐43* (associated with axonal regeneration), and *Tubb3* (neuronal identity marker) 15 min following the application of uniaxial extension, torsion, or combined extension–torsion loads. These markers were selected due to their established clinical relevance in human TBI and experimental models.^[^ ^58^, ^59^ ^]^ In parallel, we assessed Caspase‐3 activation to quantify neuronal apoptosis.

**Figure 4:**
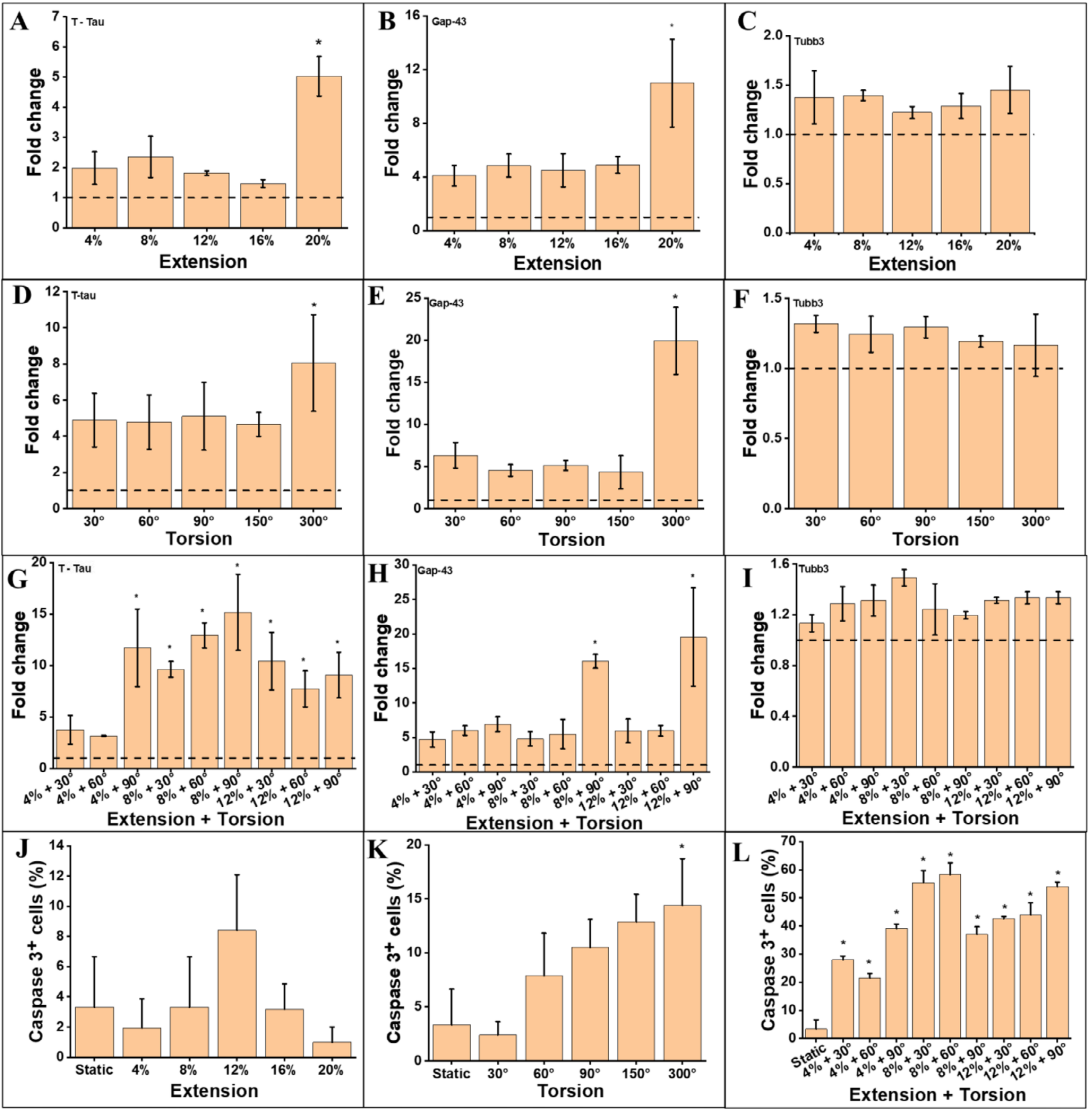
Gene expression and apoptosis profiling of CAD neurons following mechanical loading. Quantitative assessment of gene expression and apoptosis in CAD neurons following mechanical loading. RT‐qPCR was used to measure the expression levels of Mapt A,B), Gap‐43 D,E), and Tubb3 C,F,I) under uniaxial extension (4–20%), torsion (30°–300°), and combined extension–torsion (4–12% + 30°–90°) conditions. The apoptotic response was evaluated via immunostaining for cleaved Caspase‐3⁺ cells (G, H) across the same loading modalities. The dotted horizontal line denotes baseline values in static (unloaded) controls. Data represent mean ± SD from three independent biological replicates. ^*^ *p* < 0.05 versus static control.

*Mapt* expression increased in a load‐dependent manner across all mechanical stress types. A significant upregulation (p < 0.05) was observed at 20% extension (**Figure** **4**A) and at torsion angles of 300° (Figure 4D), reaching up to ≈2.5–3‐fold relative to static controls. Under combined loading, all conditions except the lowest (4% + 30° and 4% + 60°) significantly upregulated *Mapt* expression (p < 0.05, Figure 4G), with the highest induction observed at 12% + 90°, consistent with severe mechanical stress. This result is consistent with previous reports that Tau (encoded by *Mapt*) is rapidly released or upregulated following axonal degeneration and neurotrauma.^[^ ^60^ ^]^ The alignment between *Mapt* gene expression and biosensor‐detected T‐Tau protein levels in our system confirms that the NIOC platform reliably captures molecular correlates of mechanical injury. Notably, *Mapt* upregulation also mirrors the Caspase‐3 trends in Figure 4I, suggesting transcriptional and apoptotic responses are linked in the injury cascade.

*Gap‐43* expression—an established marker of axonal growth and regeneration—was significantly upregulated under high strain conditions: 20% extension (Figure 4B), 300° torsion (Figure 4E), and both 8% and 12% extension combined with 90° torsion (Figure 4H). These findings suggest that mechanical insult may trigger reparative programs in addition to injury cascades. This dual response mirrors in vivo TBI pathophysiology, where surviving neurons activate *Gap‐43* as part of a regeneration attempt.^[^ ^61^ ^]^ Interestingly, *Gap‐43* increases were not strictly correlated with Caspase‐3 activation, indicating that regenerative and apoptotic pathways may be independently modulated in response to the magnitude and complexity of mechanical deformation. These data contribute to growing evidence that regeneration attempts may begin early, even in parallel with injury‐induced apoptosis.

The expression of *Tubb3* remained stable across all loading conditions (Figure 4C,F,I), confirming the preservation of general neuronal identity. It supports the immunofluorescence observations of sustained MAP2 expression in Figures 2 and 3, and rules out general cell‐type loss as the primary cause of transcriptional changes observed for *Mapt* and *Gap‐43*. Maintaining of *Tubb3* expression provides internal validation that the applied mechanical stress did not globally suppress neuronal markers, reinforcing the specificity of observed gene regulation.

A comprehensive heat map summarizing fold changes in gene expression across all loading modalities is provided in Figure S9 (Supporting Information). The map illustrates the coordinated upregulation of *Mapt* and *Gap‐43* in response to increasing mechanical stress complexity, particularly under combined extension–torsion conditions, while *Tubb3* remains relatively stable, reinforcing the specificity of injury‐responsive transcriptional dynamics.

Quantification of cleaved Caspase‐3+ cells confirmed the apoptotic trends observed earlier. Extension loading alone did not significantly increase apoptosis (Figure 4J), whereas torsion at 300° did (Figure 4K), increasing the apoptotic fraction to ≈15% (p < 0.05 vs control). The most significant apoptotic response was observed under combined extension and torsion (Figure 4l), where Caspase‐3+ cells reached ∼25%, aligning with earlier IF results (Figure 3). This data reinforces the synergistic impact of multiaxial loading on neuronal death, as previously observed in vivo.^[^ ^52^ ^]^ Combined mechanical stresses better simulate conditions of falls, whiplash, or blast injuries, where tissues are subjected to rotational and stretching forces simultaneously. The alignment between Caspase‐3 activation and *Mapt* expression further suggests an interdependent relationship between early cytoskeletal disruption and apoptotic signaling, while *Gap‐43* provides insight into concurrent repair dynamics.

The results provide transcriptomic and cellular‐level validation of the injury patterns characterized in Figures 2 and 3. The upregulation of Mapt and increased Caspase‐3 activation under high mechanical loads confirm the load‐sensitive nature of axonal injury and apoptotic initiation. These responses closely mirror clinical observations where T‐Tau levels and apoptotic markers increase following concussive and sub‐concussive impacts. The concurrent elevation of Gap‐43 highlights the initiation of compensatory repair mechanisms, suggesting that neuronal responses to injury are not unidirectional but instead involve parallel damage and regeneration programs. Importantly, the results reveal that different mechanical loading modes elicit distinct molecular responses. Extension and torsion independently increased Mapt and Gap‐43 expression only at their uppermost magnitudes, whereas combined loading consistently triggered significant upregulation of injury markers and apoptosis. This observation supports the hypothesis that the severity of the injury is dictated not only by load magnitude but also by its geometric complexity, which is often underrepresented in traditional 2D in vitro models. Furthermore, the alignment between apoptotic (Caspase‐3+) and transcriptional (Mapt, Gap‐43) readouts under combined loading underscores the robustness of these markers for stratifying mechanical injury responses in 3D neuronal systems. Minor deviations from perfect monotonicity in Caspase‐3 levels across certain load points (e.g., 12% + 30° vs 8% + 30°) likely reflect intrinsic biological variability in apoptotic activation rather than inaccuracies in the applied mechanical inputs.

### 2.5 Ultrasensitive Biosensors Enable Near Real‐Time NFL and T‐Tau Biomarker Detection

Scanning electron microscopy (SEM) was utilized to assess the morphological progression of surface modification on the gold (Au) working electrode (WE) throughout the biosensor fabrication process. In its native state, the Au WE presented a smooth and featureless topology. Following the formation of a self‐assembled monolayer (SAM) using alkanethiol chemistry, confirmed by Fourier Transform Infrared (FTIR) spectroscopy and detailed in our recent works,^[^ ^62^, ^63^ ^]^ no apparent change in surface texture was observed, suggesting that the SAM formed a conformal, nanometric layer that uniformly coated the gold substrate. Energy‐dispersive X‐ray spectroscopy (EDS) further confirmed changes in elemental composition, indicative of chemical modification, even though SEM could not resolve topographical distinctions at this stage. Upon activation with 1‐ethyl‐3‐(3‐dimethylaminopropyl) carbodiimide (EDC) and N‐hydroxysuccinimide (NHS) and conjugation of specific monoclonal antibodies against NFL and T‐Tau, the WE surface exhibited well‐dispersed spherical nano‐ and micro‐scale structures—clearly visible in SEM—consistent with successful antibody immobilization. This provided direct structural evidence of immuno‐functionalization, confirming the biosensor readiness for target‐specific binding (Figure S5, Supporting Information). The completed biosensor was electrochemically characterized using differential pulse voltammetry (DPV) to monitor the current response to redox probe interactions at each stage of modification. The electrochemical system included a three‐electrode configuration: a gold WE for recognition and signal transduction, a carbon counter electrode (CE) for charge transfer, and a silver/silver chloride (Ag/AgCl) reference electrode (RE) for stable potential control. DPV was performed in a 4 mM ferro/ferricyanide solution to measure electron transfer resistance resulting from surface modifications.

**Figure 5:**
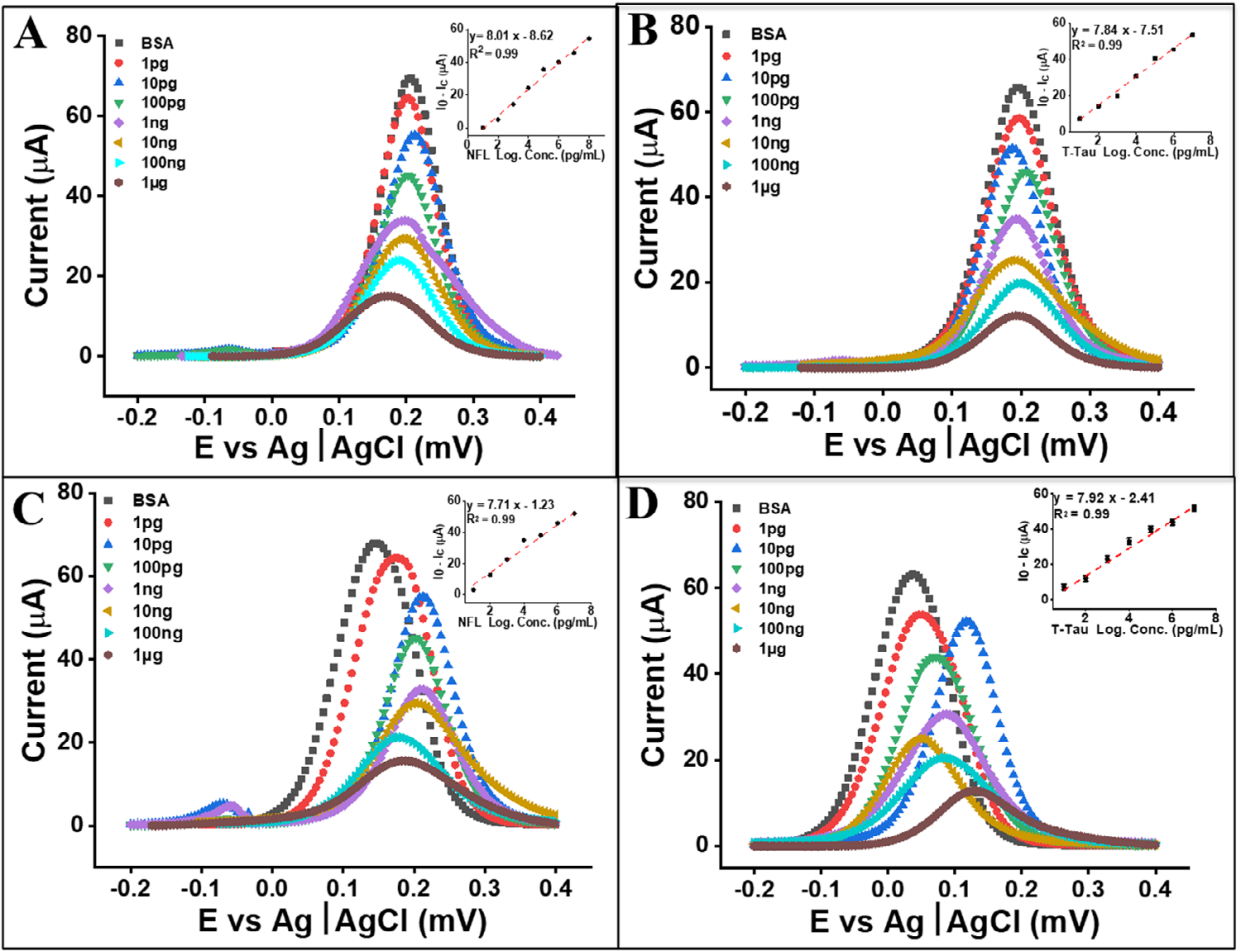
Calibration of electrochemical immuno‐biosensors for the quantitative detection of Total Tau (T‐Tau) and Neurofilament Light (NFL) proteins in buffer and culture media. (A, B) Calibration curves in phosphate‐buffered saline (PBS) demonstrate a linear response for T‐Tau A) and NFL B) across a concentration range of 100 fg mL^−1^ to 1 µg mL^−1^. (C, D) Calibration curves in DMEM culture medium confirm comparable sensitivity and linearity for T‐Tau C) and NFL D) under complex biological conditions. For each condition, the current signal (Ic) was recorded and compared to the background current from a BSA‐blocked electrode (I_0_), with the difference (I_0_–Ic) plotted to determine absolute response. Insets display linear regression fits with R^2^ = 0.99 for all sensors in both media. All measurements were performed in triplicate (n = 3), and data are presented as mean ± SEM. Reproducibility and inter‐sensor variability for this electrochemical platform have been validated previously.^[^ ^64^ ^]^

The bare gold (Au) electrode exhibited a peak DPV current of 115 µA, indicating fast electron transfer kinetics. After SAM formation, the peak current declined to 98 µA, confirming the creation of a molecular barrier that partially restricted redox species access to the electrode surface. Conjugation of the antibody further suppressed the current, dropping to 91.7 µA for NFL and 81.6 µA for T‐Tau, reflecting the increased steric hindrance and decreased conductivity associated with the antibody layers. Subsequent bovine serum albumin (BSA) blocking, which capped residual reactive sites, yielded additional decreases in signal to 75.7 µA and 72.2 µA for NFL and T‐Tau, respectively. Finally, upon exposure to 10 ng/mL of the respective target proteins in phosphate buffer saline (PBS), the current dropped significantly—reaching 32.7 µA for NFL and 28.7 µA for T‐Tau—confirming antigen–antibody binding and validating signal generation due to specific protein recognition (**Figure** **5**A, B). Electrochemical characterization of the SAM‐modified electrodes (Figure S6, Supporting Information) confirmed diffusion‐controlled redox kinetics, supporting stable and reproducible performance suitable for biosensing applications in confined microfluidic environments.

To establish calibration performance, a dilution series of T‐Tau and NFL proteins ranging from 1 µg mL^−1^ to 0.1 pg mL^−1^ was prepared in both PBS and cell culture medium (DMEM with serum). In both media, the sensors displayed a dose‐dependent decrease in peak current, reflecting the progressive binding of analyte to immobilized antibodies. In PBS, the T‐Tau biosensor showed a strong linear relationship between current and concentration, described by the equation Y = 7.84X – 7.51 with R^2^ = 0.99. The NFL biosensor followed a similar linear trend, Y = 8.01X – 8.62, also with R^2^ = 0.99. These responses validate the electrochemical mechanism of signal suppression due to increased insulating layers formed by protein binding. When tested in DMEM medium, which contains serum proteins and electrolytes, both biosensors retained excellent linearity. The calibration curve for T‐Tau in DMEM was Y = 7.92X – 2.41 (R^2^ = 0.99), while the NFL biosensor showed Y = 7.71X – 1.23 (R^2^ = 0.99) (Figure 5C,D). Although the baseline signal was slightly elevated due to matrix complexity, the high correlation and consistent slope demonstrate that the biosensors are highly robust and retain sensitivity and specificity in complex biological fluids. These results are particularly important, as many prior biosensors suffer from matrix interference when transitioning from buffer to serum or media environments. This sensing platform has been validated in prior work for TBI biomarker detection, including direct ELISA comparisons in complex matrices ^[^ ^64^ ^]^.

Compared to previously reported electrochemical biosensors, our system achieves femtogram‐per‐milliliter sensitivity, which is well below the lowest clinically reported concentrations of T‐Tau and NFL in serum and CSF (see Table S4, Supporting Information). Importantly, the design was not aimed at achieving the absolute lowest LoD in literature, but rather at ensuring clinically relevant sensitivity while maintaining a broad dynamic range to cover early‐ to late‐phase biomarker levels in TBI. This balance was achieved by optimizing electrode geometry, probe density, and electrochemical interrogation parameters, enabling accurate quantification across orders of magnitude without signal saturation. The single‐step functionalization strategy further ensures uniform probe immobilization and high reproducibility (see Table S3, Supporting Information), which are often challenging in multi‐step schemes. The slope of the calibration curves was only marginally attenuated in DMEM compared to PBS, despite the protein‐rich, electrochemically complex background of serum‐supplemented media, indicating minimal nonspecific adsorption and signal degradation due to the optimized blocking and surface chemistry. These findings validate the biosensors as robust, reproducible tools for quantitative detection of T‐Tau and NFL, with retained performance across matrices, supporting their translational relevance and suitability for near real‐time monitoring in cell culture and preclinical injury models.

### 2.6 Finite Element Modeling Validates Load Transmission and Injury Correlation

To verify that the applied mechanical loads were effectively transmitted to the neuronal layer within the PDMS tube, we performed finite element modeling (FEM) simulations of the experimental loading conditions using ABAQUS. The PDMS tube was modeled as a nonlinear hyperelastic body using the Ogden material model. Meshes were generated using C3D20H solid elements, and the bottom face of the tube was fully constrained, while the top face was subjected to displacement‐controlled loading via a reference point coupling. The best‐fit Ogden parameters (μ = 19.14 kPa, α = 1.5, D = 3×10^−^⁸) were obtained by matching the model's axial force response to experimental tension data (see detail in Figures S1–S4, Supporting Information).

Principal stress analysis showed that under uniaxial extension, the maximum first principal stress increased from ≈0.26 MPa at 4% strain to ≈1.01 MPa at 16% strain, reaching 1.27 MPa at 20% extension. Under torsion, principal stress values scaled with rotation angle, increasing from 28.7 kPa at 30° to 288 kPa at 300° twist (Table S1, Supporting Information). For combined extension and torsion, the highest stress values were observed at 12% + 90°, yielding a stress of ≈787 kPa, which closely paralleled experimental biomarker and apoptotic responses. Interestingly, loading combinations in the midrange (e.g., 8% + 60°) resulted in intermediate principal stress (≈525 kPa), aligning with moderate Caspase‐3 activation and gene upregulation. FE simulations of the PDMS tube under axial extension (4–20%), torsion (30°–300°), and combined loading reproduced inner‐wall maximum principal strains spanning ≈0.10–0.25, consistent with deformation levels linked to axonal injury in published tissue stretch experiments and FE head models of TBI ^[^ ^27^, ^52^, ^53^, ^54^ ^]^.

These simulations confirm that the biomechanical inputs applied externally result in a predictable and spatially resolved internal stress profile that correlates with injury biomarkers such as T‐Tau and NFL. Notably, the first principal stress emerged as a consistent frame‐invariant scalar metric of mechanical insult. A stress‐based damage metric (σ̂, Euclidean norm of principal stresses) revealed that stress magnitude alone could stratify injury severity across different loading geometries. Cases with σ̂ > 0.75 MPa consistently aligned with conditions that induced strong apoptotic and biomarker responses, establishing a quantitative framework for linking mechanical stimuli to neuronal fate. The numerical model validated the experimental approach by demonstrating that extension, torsion, and combined mechanical loads were successfully transferred to the neuron‐laden surface of the cylindrical construct. This highlights the NIOC platform's reliability in replicating physiologically relevant mechanical environments in vitro, reinforcing its utility for high‐fidelity modeling of TBI.

### 2.7 Acute Biomarker Trajectories Mirror Transcriptional and Apoptotic Responses

**Figure 6:**
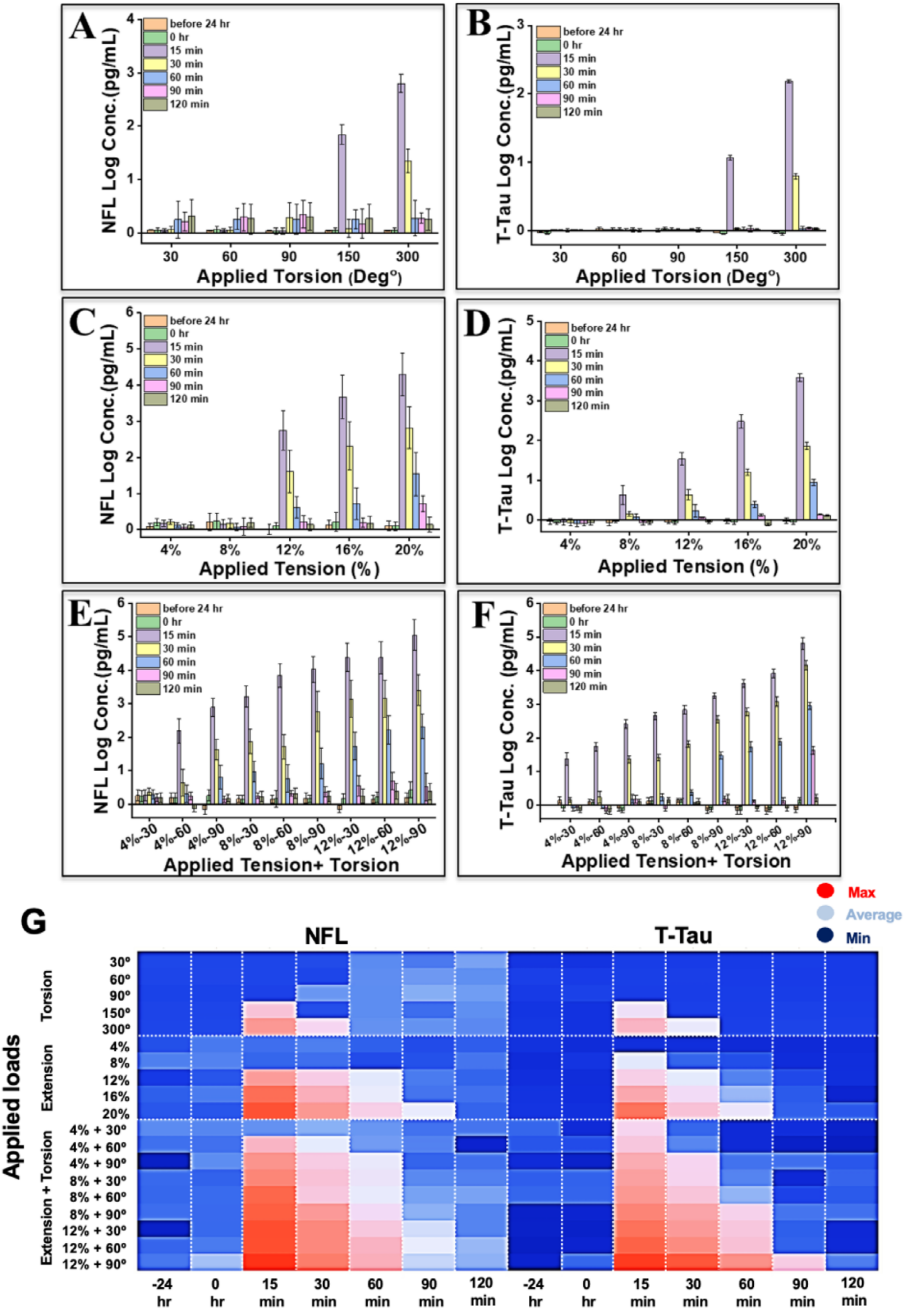
Near real‐time quantification of T‐Tau and NFL release following mechanical loading of 3D‐cultured CAD neurons. Time‐course analysis of NFL A–C) and T‐Tau D–F) concentrations in conditioned medium collected after applying (A, B) torsion (30°–300°), (C, D) uniaxial extension (4%–20%), and (E, F) combined extension (4%–12%) with torsion (30°–90°). Each data point represents mean ± SEM from five independent experiments (n = 5). Protein concentrations were measured at 15, 30, 60, 90, and 120 min post‐loading using electrochemical immuno‐biosensors calibrated as shown in Figure 5. Peak T‐Tau values fell within the low‐to‐mid pg·mL^−1^ to low‐hundreds pg·mL^−1^ band at 15 min, while NFL rose later and extended into the tens‐to‐hundreds pg·mL^−1^ range—closely matching clinically reported acute blood concentrations for mTBI–moderate TBI (Table S4, Supporting Information). Concentrations gradually declined over the 2 h post‐injury period. Near real‐time quantification of T‐Tau and NFL release and corresponding transcriptional responses following mechanical loading of 3D‐cultured CAD neurons. G) Heat map summarizing T‐Tau and NFL levels at 15 min post‐loading across all mechanical conditions. Data are normalized to static controls and highlight biomarker‐specific thresholds of response under increasing load complexity, with the highest expression observed under combined loading conditions.

To assess dynamic neuronal injury in response to mechanical stress, the concentrations of T‐Tau and NFL were quantified in conditioned medium using calibrated biosensors. These proteins are well‐established in both experimental and clinical TBI contexts as fluid‐phase biomarkers of axonal disruption and neuronal degeneration.^[^ ^62^ ^]^ Their elevation in cerebrospinal fluid (CSF) and serum correlates with injury severity, clinical outcomes, and progression toward chronic neurodegenerative conditions such as chronic traumatic encephalopathy (CTE) and post‐concussion syndrome. Media was sampled at multiple timepoints after mechanical loading to track the acute release profile of each protein. Consistent across all loading modalities, T‐Tau and NFL levels peaked at 15 min post‐load application, followed by a progressive decrease over the next 2 h (**Figure** **6**). This early burst pattern is reminiscent of clinical studies in which serum T‐Tau rises within minutes to hours of injury and NFL levels remain elevated from hours to days, depending on injury severity ^[^ ^65^ ^]^.

Importantly, the degree of biomarker elevation was proportional to the intensity and complexity of the mechanical load applied. For torsional loading, protein levels remained low below 90°, but increased markedly above 150° of twist (Figure 6A,B). Under uniaxial extension, T‐Tau concentrations increased significantly above 8% strain, while NFL concentrations rose only at the highest strain tested (≥12%), suggesting that T‐Tau may be a more sensitive early indicator of axonal stress (Figure 6C,D). This finding supports previous literature showing that torsional or rotational forces are especially damaging to axons, where shear deformation is known to cause cytoskeletal breakdown and membrane permeability changes.^[^ ^66^ ^]^ The trends are also consistent with studies reporting that T‐Tau correlates with microtubule damage and dendritic beading, while NFL is more strongly associated with cytoskeletal rupture and axonal transection ^[^ ^67^ ^]^.

The most pronounced biomarker elevations were observed under combined mechanical loading conditions, particularly at 12% extension and 90° torsion (Figure 6E,F). In these cases, both T‐Tau and NFL concentrations exceeded the levels induced by any single‐axis load, highlighting the synergistic impact of multiaxial loading. This effect is consistent with earlier findings of increased apoptotic activity (Caspase‐3+) and the upregulation of injury‐responsive genes such as Mapt and Gap‐43 (Figures 3 and 4), suggesting a coordinated cellular response to complex biomechanical stress. The temporal profile of protein release closely mirrored the transcriptional responses captured under the same conditions. T‐Tau secretion coincided with elevated Mapt expression, reflecting early microtubule destabilization, while increased NFL levels paralleled Gap‐43 activation, indicative of axonal remodeling and injury repair. This alignment between mRNA and protein signals supports the notion that transcriptional priming contributes to downstream protein release following cytoskeletal strain. The simultaneous activation of Caspase‐3 further reinforces a phased model of neuronal response—where early cytoskeletal injury leads to molecular disassembly, apoptotic signaling, and attempted structural compensation. To complement the primary kinetic and mechanical load–response data, we compiled the temporal trajectories of T‐Tau and NFL across all loading modalities into an integrated comparative format (Figure S8, Supporting Information). This composite representation captures both the time‐dependent dynamics and the load‐specific scaling of biomarker release, reinforcing the distinct sensitivity thresholds and kinetics of each marker under varying mechanical insults.

Notably, the 15‐min T‐Tau peak under combined loading coincides temporally with the upregulation of Mapt transcripts and supports the hypothesis that transcriptional activation and protein release are tightly coupled during the acute post‐injury phase. This observation is in line with neuropathological findings from both rodent and swine models of TBI, where combined mechanical loads result in diffuse axonal injury, subdural hemorrhages, and glial activation not seen in linear loading alone.^[^ ^68^ ^]^ Human imaging studies using diffusion tensor imaging (DTI) and Susceptibility‐Weighted Imaging (SWI) have also confirmed that rotational and oblique loading patterns are associated with greater white matter disruption than linear impact ^[^ ^69^ ^]^.

A biosensor‐based heat map (Figure 6G) further illustrates the condition‐specific expression profiles of T‐Tau and NFL across the full mechanical load matrix. Consistent with the time‐course data, protein levels were modest under low‐strain torsion or extension, but markedly elevated under high‐strain or combined loading. This representation visually emphasizes the sharp biomarker response threshold triggered by multiaxial loading, supporting the use of multiplex biosensors for injury stratification in mechanically dynamic environments.

### 2.8 Translational Potential and Future Directions

The NIOC platform represents a next‐generation organ‐on‐chip system for modeling central nervous system (CNS) trauma under physiologically relevant mechanical loading conditions. By integrating programmable, multiaxial loading with electrochemical biosensors, NIOC enables near real‐time, label‐free quantification of axonal injury biomarkers such as total tau (T‐Tau) and neurofilament light chain (NFL) with picogram‐level sensitivity. This capability fundamentally distinguishes the platform from conventional in vitro systems, which typically rely on static, end‐point assays such as ELISA, western blotting, or immunostaining—methods that require large sample volumes, pooling, and cannot resolve temporal dynamics of injury response.

Unlike traditional stretch or compression devices that apply uniaxial stress, the NIOC platform delivers multiaxial loading—including extension, torsion, and their combinations—mirroring the complex forces implicated in human TBI from sports impacts, vehicular accidents, and blast injuries. The cylindrical microfluidic design provides uniform strain distribution, as confirmed through finite element modeling (FEM), enabling precise and reproducible load delivery. These multiaxial inputs allow stratification of neuronal injury severity based on load magnitude and geometry, a critical advancement for modeling diffuse axonal injury and other heterogeneous TBI phenotypes.

Critically, the platform integrates electrochemical biosensors directly into the microfluidic environment. These sensors eliminate the need for destructive sampling or signal amplification and are uniquely positioned to monitor early‐stage biomarker secretion within confined microenvironments. Compared to conventional ELISA, they require significantly lower sample volumes, retain analytical resolution in serum‐containing media, and capture dynamic injury signatures in the acute post‐injury phase. Our sensors were benchmarked against Simoa technology and demonstrated equivalent sensitivity, validating their utility in detecting clinically relevant biomarkers such as T‐Tau and NFL. This makes the system highly suitable for continuous monitoring of sub‐concussive and concussive events.

Importantly, the integration of genomic and proteomic data—spanning qPCR quantification of Mapt and Gap‐43, Caspase‐3 immunostaining, and biosensor readouts—provides a holistic view of mechanobiological injury. Our results uncover a cascade of events triggered by load‐dependent cytoskeletal stress, including transcriptional activation, protein release, and apoptosis. The system revealed distinct biomarker trajectories across different loading conditions, with combined extension–torsion producing the strongest injury response. These findings closely mirror neuropathological observations in TBI patients and animal models, where multiaxial stresses are associated with higher rates of axonal shearing, cytoskeletal disruption, and glial activation.

While several biosensing strategies in the literature have reported LoDs marginally lower than ours, such ultra‐high sensitivity often comes at the expense of dynamic range and reproducibility. In contrast, our single‐step functionalization strategy enables uniform probe immobilization across multiple electrodes, reducing inter‐sensor variability and supporting multiplexing. By integrating this robust biosensing layer into the NIOC platform, we provide a system that is sensitive enough for clinically relevant biomarker concentrations, maintains a broad detection range, and is scalable for translational neurotrauma monitoring.

The translational potential of NIOC is significant. The platform could support biomarker threshold refinement for sideline concussion assessments, risk stratification, and return‐to‐play decisions in sports medicine. Its ability to resolve early molecular events before overt cell death opens opportunities for therapeutic testing in narrow intervention windows. Additionally, the system's compatibility with high‐throughput screening and longitudinal analysis positions it as a valuable tool for neuroprotective drug discovery.

The inclusion of finite element modeling further strengthens the system's predictive power. Using a hyperelastic Ogden formulation, we achieved an accurate simulation of internal stress distributions that aligned with biological outputs. Because the numerical framework depends only on input strain and calibrated material properties, it can be extended to other geometries and cell types without the need for experimental re‐validation. Future enhancements could incorporate viscoelasticity, time‐dependent loading, or region‐specific mechanical heterogeneity to mimic in vivo complexities more faithfully.

Although the PDA coating demonstrated stability under the high‐magnitude extension and torsion loads applied here, long‐term cyclic mechanical loading was not evaluated. For chronic neurotrauma models that involve repeated strain over days to weeks, systematic multi‐cycle mechanical stability testing of the coating would be an important next step. The literature indicates that PDA–PDMS coatings can remain intact for extended periods under static or flow conditions, but verifying this under repetitive loading will further strengthen confidence in the platform's suitability for long‐duration applications.

Moving forward, future iterations of the NIOC platform will incorporate primary neuronal cells and additional CNS cell types such as astrocytes, microglia, and endothelial cells to capture both primary and secondary injury mechanisms. This will allow modeling of neuroinflammatory cascades, blood–brain barrier disruption, and glial scarring—hallmarks of moderate and severe TBI progression. The integration of perfusable vasculature and immune responsiveness will further enhance the fidelity and translational utility of the platform.

## 3 Conclusion

We present the Neuron‐Injury‐on‐a‐Chip (NIOC) platform, a biointegrated system that unites programmable 3D mechanical loading with real‐time electrochemical biosensing to decode neuronal responses to physiologically relevant mechanical insults. By leveraging a cylindrical microfluidic architecture seeded with differentiated neurons and functionalized with polydopamine, NIOC enables uniform application of controlled extension, torsion, and combined multiaxial loading. Finite element modeling confirmed precise load transmission, while multiplexed biosensors provided near real‐time, label‐free detection of axonal injury biomarkers—T‐Tau and NFL—with picogram sensitivity and minimal sample volume. Transcriptomic profiling of Mapt and Gap‐43, along with Caspase‐3 immunostaining, revealed load‐specific gene and protein trajectories, capturing the early molecular dynamics of mechanobiological injury and neurodegeneration.

Clinically, the system offers a powerful surrogate to model mild‐to‐moderate traumatic brain injury (TBI), diffuse axonal injury (DAI), and sub‐concussive trauma—conditions often elusive to neuroimaging yet predictive of long‐term cognitive decline. The ability to stratify injury responses by loading geometry, capture early biomarker kinetics, and link mechanical stimuli to transcriptional and apoptotic signaling closely aligns with current translational priorities in neurotrauma diagnostics. Moreover, the platform's modularity, high temporal resolution, and compatibility with human‐relevant biomarkers position it for therapeutic screening, safety threshold mapping, and the development of precision diagnostics for CNS injury. Future iterations incorporating astrocytes, microglia, and vascular elements will enable comprehensive modeling of both primary and secondary injury cascades, advancing NIOC as a next‐generation tool for dissecting CNS trauma and accelerating biomarker‐guided interventions.

## 4 Experimental Section

### Fabrication of PDMS Cylindrical Tubes

PDMS was prepared by mixing the pre‐polymer and curing agent at a 10:1 weight ratio and degassed thoroughly in three cycles to eliminate air bubbles.^[^ ^43^ ^]^ A destructible plastic mold was used as a casting frame to form the 3D cylindrical structure. A polypropylene syringe with a 5 mm inner diameter served as the outer mold, and a centrally aligned iron rod (8 cm in length, 1.5 mm in diameter) was inserted to define the inner channel. The syringe was filled with the uncured PDMS mixture and sealed at both ends using gaskets to prevent leakage. The iron rod was inserted longitudinally through the gaskets, ensuring it remained centered within the PDMS to form a uniform axial channel. The assembly was cured in an oven at 70 °C for 2 h. Once cured, the plastic mold was carefully cut open to extract the PDMS tube, yielding a 5 mm‐long cylindrical chip with a central channel of 1.5 mm diameter.

### Seeding CAD Cells Into the Cylindrical Microchannel

The inner surfaces of the PDMS cylindrical tubes were first coated with polydopamine (PDA) using a continuous flow protocol, following established procedures. To enhance cell adhesion, a 5 mg mL^−1^ solution of rat tail collagen was injected into each tube and rotated every 30 min for uniform coating. After 2 h of incubation, the tubes were rinsed three times with culture medium to remove excess collagen. A suspension of CAD (Cath.a‐differentiated) neuronal cells was prepared at a concentration of 75 million cells mL^−1^. ≈100 µL of the cell suspension was introduced into each microchannel through a sterile plastic cylindrical connector. The tubes were incubated for 2 h in a horizontal position to allow initial cell adhesion to the lower inner wall. Subsequently, the tubes were rotated 180° and incubated for an additional 2 h to achieve uniform seeding along the inner circumference. Following the attachment, a fresh culture medium was flushed through the microchannel to remove any non‐adherent cells. The seeded tubes were maintained under standard incubation conditions for 5 days, with media replenishment every 12 h to support neuronal growth and viability within the 3D microfluidic environment.

### Immunofluorescent Staining

CAD cells were fixed with 4% paraformaldehyde for 10 min and permeabilized using 0.05% Triton X‐100 for 10 min. Immunofluorescent staining was performed in the dark using a rabbit anti‐MAP2 (microtubule‐associated protein 2) primary antibody (1:200), incubated overnight at 4 °C. This was followed by staining with a donkey anti‐rabbit Alexa Fluor 594 secondary antibody (1:1000) for 2 h at room temperature.^[^ ^43^ ^]^ Subsequently, cells were stained with Alexa Fluor 647‐conjugated monoclonal Caspase‐3 antibody (1:50), a marker of apoptosis, along with an F‐actin probe (1:100), and incubated for 1 h at room temperature. Fluorescent images were acquired using confocal microscopy, and the percentage of caspase‐3–positive cells was quantified using Image‐Pro Plus software.

### Quantitative Real‐Time Polymerase Chain Reaction (qRT‐PCR)

Total RNA was extracted from cultured CAD cells using the PureLink™ RNA Mini Kit according to the manufacturer's instructions. Complementary DNA (cDNA) was synthesized from the isolated RNA using the High‐Capacity RNA‐to‐cDNA™ Kit and a thermal cycler, following standard protocol. The resulting cDNA was combined with TaqMan™ Fast Advanced Master Mix and target‐specific TaqMan Gene Expression Assays. Quantitative real‐time PCR (qRT‐PCR) was performed using a compatible qRT‐PCR system. Target genes included Mapt (Mm00521988_m1), Tubb3 (Mm00727586_s1), and Gap43 (Mm00500404_m1), with Gapdh (Mm99999915_g1) used as the endogenous control for normalization. All expression data were analyzed using the ΔΔCt method and reported as fold change relative to static controls (CAD cells cultured in the microfluidic chip without mechanical loading).

### Biosensor Fabrication and Device Assembly

A three‐electrode configuration was employed for biosensing, consisting of a gold (Au) working electrode (WE), an Au counter electrode (CE), and a platinum (Pt) reference electrode (RE), following fabrication protocols detailed in the previous work.^[^ ^62^ ^]^ To functionalize the WE for selective detection of T‐Tau and NFL, self‐assembled monolayer (SAM) modification and monoclonal antibody immobilization were performed. Briefly, Au electrodes were immersed in 5 mM 11‐mercaptoundecanoic acid (11‐MUA, 95% purity) prepared in ethanol and incubated for 24 h to form alkanethiol SAMs on the electrode surface.^[^ ^70^ ^]^ Following SAM formation, electrodes were treated with a mixture of 50 mm N‐(3‐dimethylaminopropyl)‐N′‐ethylcarbodiimide hydrochloride (EDC, ≥98%) and 50 mm N‐hydroxysuccinimide (NHS, 98%) for 2 h to activate the terminal carboxylic acid groups of 11‐MUA, forming reactive NHS esters on the surface.^[^ ^71^ ^]^ This allowed for covalent attachment of 20 µL of monoclonal antibodies against T‐Tau or NFL (25 µg mL^−1^ in 10 mm PBS, pH 7.0), which were incubated on the surface for 45 min. To block non‐specific binding sites, the electrodes were subsequently treated with 0.25% bovine serum albumin (BSA) for 30 min. The resulting antibody‐functionalized electrodes were used for selective detection of T‐Tau and NFL proteins released by neuron‐laden cylindrical tissue constructs.

Individual electrochemical biosensors developed for the detection of T‐Tau and NFL were calibrated using known concentrations of the target proteins spiked in phosphate‐buffered saline (PBS) and DMEM cell culture medium. For each assay, 20 µL of T‐Tau or NFL solution was applied to the corresponding biosensor and incubated for 30 min. Following incubation, the electrode surface was rinsed with PBS and overlaid with 4 mM [K_3_Fe(CN)_6_]/[K_4_Fe(CN)_6_] redox probe solution. Differential pulse voltammetry (DPV) was then performed to generate electrochemical readouts. The potentiostat was operated from –0.4 to +0.4 V with a step potential of 0.05 V, a pulse width of 100 ms, a pulse period of 200 ms, and a pulse amplitude of 0.03 V. Current responses were recorded and plotted against antigen concentrations to generate calibration curves for each biosensor, establishing a quantitative relationship between protein concentration and DPV signal intensity.

The PDMS tube was secured at both ends using programmable grippers for extension and torsion loading. A small port positioned at the midpoint of the tube was connected via a short sampling line to a miniaturized solenoid valve, which directed aliquots of the perfusate to a CapSense electrochemical sensing chip, as previously described.^[^ ^42^ ^]^ This configuration enabled controlled, on‐demand sampling during mechanical loading without disrupting device operation, while minimizing dead volume so that the cell culture environment remained unaffected. The CapSense chip was modular and replaced between runs, and it was configured for dual‐analyte sensing using two working electrodes ^[^ ^72^ ^]^.

Axial strain and torsion were controlled using a motorized linear actuator (±0.01 mm positional accuracy, corresponding to ±0.2% strain resolution) and a stepper‐motor‐driven rotational stage (±0.1° angular accuracy, <0.5% variation in twist‐induced strain), synchronized through a microcontroller interface to ensure repeatable combined loading profiles. All mechanical calibration and testing were performed in an ISO 9001‐certified laboratory to maintain compliance with recognized quality standards. Each target strain and twist value was calibrated using optical markers on the tube surface and validated in triplicate (coefficient of variation < 3%). The programmable hardware enables identical mechanical profiles to be reproduced across experiments or scaled to different tube geometries by recalibrating the strain‐to‐displacement mapping.

### Data Analysis and Statistics

Electrochemical data obtained from the biosensors were baseline‐corrected, processed, and visualized using OriginPro 2016 (OriginLab Corporation, USA). Statistical analyses were performed using one‐way analysis of variance (ANOVA) followed by Tukey's post hoc test for multiple comparisons. A minimum of five biological replicates (n ≥ 5) was used per experimental group. Statistical significance was defined as *p* < 0.05.

### Effects of Mechanical Loading on the PDMS Tube

The mechanical response of the cylindrical PDMS tubes was evaluated using both analytical modeling and finite element method (FEM) simulations. While the analytical formulation is presented below, the FEM results—which closely match the analytical predictions—are provided in the Supplementary Materials. Since the experimental setup applied mechanical loading in a displacement‐controlled manner (for both extension and torsion), the analytical analysis was based solely on kinematic relationships, assuming the PDMS material to be isotropic, homogeneous, and incompressible. These assumptions are valid given that PDMS, like most elastomers, exhibits near‐incompressibility and lacks fiber reinforcement, rendering it effectively isotropic under deformation. This formulation enables precise estimation of internal stress and strain fields during the combined loading scenarios used in this study.

The effects of mechanical loads on the cylindrical tubes were evaluated via both an analytical model and using the finite element method (FEM). Here, we present the analytical formulation, while the results of the FEM simulation, which were equivalent to the analytical results, are reported in the Complementary Materials. Since the experiments are performed under displacement control for both applied extension and torsion mechanical loads, the analytical calculations can be performed solely based on kinematics, under the hypothesis of an isotropic, homogeneous, and incompressible material. This hypothesis is satisfied, since PDMS is, like all polymers, virtually incompressible and can be safely regarded as isotropic (no fibre reinforcement). Consider a tube of referential length L, internal radius R_int_ and external radius R_ext_, subjected to simultaneous extension and torsion. The axial deformation introduces a uniform axial stretch given by:

(1) $$\lambda =\frac{l}{L}=1+\frac{l-L}{L}=1+\varepsilon$$

where l is the deformed length of the tube, and we used the familiar nominal strain

(2) $$\varepsilon =\frac{l-L}{L}\;.$$

The torsion is applied by a twist angle ψ, resulting in a twist angle per unit reference length defined as

(3) $$\psi =\frac{\Psi }{L}\;.$$

The internal surface of the tube, coated with PDA and collagen and seeded with CAD cells, is assumed to have negligible stiffness relative to the bulk PDMS material. As such, the coating does not contribute to the mechanical response and is considered mechanically passive. Additionally, because the tube is unconstrained on its outer surface, no external pressure is applied. Consequently, there are no applied radial stresses on either the internal or external surfaces apart from the imposed axial extension and torsional loading. Given the assumptions of isotropy and near‐incompressibility for PDMS, the radial stress is also considered negligible throughout the radial domain of the material. Under these conditions, the deformation behavior of a hollow tube with internal radius R_int_ and external radius R_ext_ is kinematically equivalent to that of a solid incompressible cylinder of the same length and arbitrary radius. Therefore, established formulations for the extension‐torsion response of incompressible solid cylinders are adopted for modeling the deformation of the PDMS tubes in this study.

### Kinematics

Under simultaneous extension and torsion, characterized by an axial stretch ratio λ and a twist angle R *ψ* per unit reference length, the deformation map *ϕ* of a solid cylinder in cylindrical material and spatial coordinates is given by

(4) $$\left(r,\theta ,z\right)=\phi \;\left(R,\Theta ,Z\right)=\left(f\left(R\right),\Theta +\psi Z,\lambda \;Z\right)$$

where the radial deformation function f is derived from the deformation gradient F, represented in matrix form as detailed in ^[^ ^73^, ^74^ ^]^, as

(5) $$\left[F\right]\;\left(R,\Theta ,Z\right)=\left[\begin{array}{ccc} f^{\prime }\left(R\right) & 0 & 0\\ 0 & \frac{f\left(R\right)}{R} & \psi \;f\left(R\right)\\ 0 & 0 & \lambda \end{array}\right]$$

The incompressibility constraint imposes that J=detF=1, which, applied to Equation (5), results in

(6) $$f^{\prime }\left(R\right)f\;\left(R\right)=\frac{R}{\lambda }$$

Integrating Equation (6) yields

(7) $$\frac{1}{2}\;{\left[f\left(R\right)\right]}^{2}=\frac{1}{2}\;\frac{R^{2}}{\lambda }+\frac{1}{2}c$$

Simplifying, the radial deformation function becomes

(8) $$r=f\;\left(R\right)=\lambda^{-1/2}\;\sqrt{R^{2}+\lambda \;c}$$

For a solid cylinder under an extension or torsion load, we have f(0)=0 (the point on the axis remains unchanged on the axis throughout the deformation), therefore the constant *c* in Equation (8) must vanish and the radial deformation takes the final form

(9) $$r=f\;\left(R\right)=\lambda^{-1/2}\;R.$$

Consequently, under the simplified radial deformation $f\left(R\right)=\lambda^{-1/2}R$, the matrix form of the deformation gradient ***F*** in equation (5) reduces to

(10) $$\left[F\right]\;\left(R,\Theta ,Z\right)=\left[\begin{array}{ccc} \lambda^{-1/2} & 0 & 0\\ 0 & \lambda^{-1/2} & \psi \;R\;\lambda^{-1/2}\\ 0 & 0 & \lambda \end{array}\right]$$

### Principal Stretches and Principal Strains

To evaluate the principal stretches and the principal directions of the deformation shown in Equation (10), we evaluated the representing matrix of the right Cauchy‐Green deformation tensor $C=F^{T}.F$, i.e.,

(11) $$\left[C\right]\;\left(R,\Theta ,Z\right)=\left[\begin{array}{ccc} \lambda^{-1} & 0 & 0\\ 0 & \lambda^{-1} & \psi \;R\;\lambda^{-1}\\ 0 & \psi \;R\;\lambda^{-1} & \lambda^{2}+\psi^{2}R^{2}\lambda^{-1} \end{array}\right]$$

Extracted its eigenvalues and take their square roots to obtain the principal stretches **λ**_**α**_. We observe that the radial direction is already a principal direction, and thus the first principal stretch is the radial stretch $\lambda^{-1/2}$. The other two stretches are the square roots of the eigenvalues of the sub‐matrix

(12) $$A]=\left[\begin{array}{cc} \lambda^{-1} & \psi \;R\;\lambda^{-1}\\ \psi \;R\;\lambda^{-1} & \lambda^{2}+\psi^{2}R^{2}\lambda^{-1} \end{array}\right]$$

Which are equal to

(13) $$\begin{array}{c} \lambda_{1}=\sqrt{\frac{\lambda^{3}+\psi^{2}R^{2}+1+\sqrt{{\left(\lambda^{3}+\psi^{2}R^{2}+1\right)}^{2}-4\;\lambda^{3}}}{2\;\lambda }}\;>1,\\ \;\lambda_{2}=\sqrt{\frac{\lambda^{3}+\psi^{2}R^{2}+1-\sqrt{{\left(\lambda^{3}+\psi^{2}R^{2}+1\right)}^{2}-4\;\lambda^{3}}}{2\;\lambda }}\;<1. \end{array}$$

These stretches, evaluated at R=R_int_, describe the deformation in the plane tangent to any generator of the inner surface of the tube, under any combined axial stretch λ and unit twist angle *ψ*. Note that we always have $\lambda_{1}>1$ and $\lambda_{2}<1$. In terms of principal nominal (Biot) strains, this translates into $\varepsilon_{1}=\lambda_{1}-1>0$ and $\varepsilon_{2}=\lambda_{2}-1<0$. The presence of a negative nominal strain is consistent with the general behavior of materials under torsion and is further enforced by the incompressibility constraint. Finite element simulations incorporating these assumptions were used to calculate the internal stress and strain distributions within the tube. The simulation outcomes confirmed that the externally applied extension and torsion loads are effectively transmitted to the interior of the cylindrical structure where neurons are cultured, corroborating the analytical predictions and experimental results.

The Finite Element analysis was conducted using ABAQUS to simulate the mechanical stress distribution within the PDMS tube under extension, torsion, and combined loading. The model used C3D20H hybrid elements and implemented the Ogden hyperelastic material formulation with parameters *μ* = 19.14 kPa, *α* = 1.5, and *D* = 3 × 10^−^⁸ kPa^−1^, derived by fitting experimental tension data. The bottom face of the tube was fixed, and loading was applied at the top surface via a kinematic coupling. A Python automation script was employed to sequentially simulate all experimental loading cases. Assuming that the inner coating (PDA/collagen/neurons) contributed negligible stiffness, strain values at the PDMS inner wall were taken as representative of the strain experienced by neuronal cells. Additional simulation results and stress data are provided in Supplementary Information (Table S1, Supporting Information).

## Conflict of Interest

The authors declare no conflict of interest.

## Author Contributions

S.K., AP.S performed biosensor fabrication, electrochemical measurements, designed and fabricated the 3D microfluidic platform, conducted experimental data analysis, and contributed to manuscript drafting. M.F.A., A.H., and L.S. developed the analytical model and conducted finite element simulations. K.W.Y. performed cell culture, immunofluorescence staining, and imaging. K.W.Y assisted with cell seeding and qPCR analysis. AP.S., O.K., K.W., and A.R. supported data processing and statistical analysis. S.F. contributed to extension–torsion modeling and analytical validation. K.K. and A.S. supervised experimental design and biosensor assay optimization. A.S.N. conceived the study, secured funding, supervised all aspects of the project, oversaw data analysis and interpretation, and finalized the manuscript.

## Supporting information



Supporting Information

## Data Availability

The data that support the findings of this study are available from the corresponding author upon reasonable request.

## References

[1] J. S. Easter , J. S. Haukoos , W. P. Meehan , V. Novack , J. A. Edlow , JAMA, J. Am. Med. Assoc. 2015, 314, 2672. 10.1001/jama.2015.16316 26717031

[2] S. Y. Ng , A. Y. W. Lee , Frontiers in Cellular Neuroscience 2019, 13, 528. 31827423 10.3389/fncel.2019.00528 PMC6890857

[3] A. S. Jagoda , J. J. Bazarian , J. J. Bruns Jr , S. V. Cantrill , A. D. Gean , P. K. Howard , J. Ghajar , S. Riggio , D. W. Wright , R. L. Wears , Journal of Emergency Nursing 2009, 35, 5. 19285163 10.1016/j.jen.2008.12.010

[4] A. L. Sharp , G. Nagaraj , E. J. Rippberger , E. Shen , C. J. Swap , M. A. Silver , T. McCormick , D. R. Vinson , J. R. Hoffman , Academic Emergency Medicine 2017, 24, 22. 27473552 10.1111/acem.13061

[5] J. J. Bazarian , P. Biberthaler , R. D. Welch , L. M. Lewis , P. Barzo , V. Bogner‐Flatz , P. G. Brolinson , A. Büki , J. Y. Chen , R. H. Christenson , Lancet Neurol. 2018, 17, 782. 30054151 10.1016/S1474-4422(18)30231-X

[6] I. Thomas , A. M. Dickens , J. P. Posti , E. Czeiter , D. Duberg , T. Sinioja , M. Kråkström , I. R. Retel Helmrich , K. K. Wang , A. I. Maas , Nat. Commun. 2022, 13, 2545. 35538079 10.1038/s41467-022-30227-5 PMC9090763

[7] L. Gaetani , K. Blennow , P. Calabresi , M. Di Filippo , L. Parnetti , H. Zetterberg , Journal of Neurology, Neurosurgery & Psychiatry 2019, 90, 870. 30967444 10.1136/jnnp-2018-320106

[8] M. Syk , E. Tornvind , M. Gallwitz , D. Fällmar , Å. Amandusson , H. Rothkegel , T. Danfors , M. Thulin , A. J. Rasmusson , S. Cervenka , Translational Psychiatry 2024, 14, 304. 39048548 10.1038/s41398-024-03021-8 PMC11269634

[9] N. Pankratova , M. Jović , M. E. Pfeifer , RSC Adv. 2021, 11, 17301. 34094508 10.1039/d1ra00589h PMC8114542

[10] I. Hossain , N. Marklund , E. Czeiter , P. Hutchinson , A. Buki , Brain and Spine 2024, 4, 102735. 38510630 10.1016/j.bas.2023.102735 PMC10951700

[11] D.‐S. Kim , G.‐W. Kim , Brain & Neurorehabilitation 2024, 17, 8. 10.12786/bn.2024.17.e22 PMC11621674 39649708

[12] Y. Li , L. Zhang , S. Kallakuri , A. Cohen , J. M. Cavanaugh , Journal of the Neurological Sciences 2015, 359, 280. 26671128 10.1016/j.jns.2015.08.035

[13] E. Bar‐Kochba , M. T. Scimone , J. B. Estrada , C. Franck , Sci. Rep. 2016, 6, 30550. 27480807 10.1038/srep30550 PMC4969749

[14] P. R. Williams , B.‐N. Marincu , C. D. Sorbara , C. F. Mahler , A.‐M. Schumacher , O. Griesbeck , M. Kerschensteiner , T. Misgeld , Nat. Commun. 2014, 5, 5683. 25511170 10.1038/ncomms6683

[15] L. Galluzzi , K. Blomgren , G. Kroemer , Nat. Rev. Neurosci. 2009, 10, 481. 19543220 10.1038/nrn2665

[16] Y. Mi , G. Qi , F. Vitali , Y. Shang , A. C. Raikes , T. Wang , Y. Jin , R. D. Brinton , H. Gu , F. Yin , Nature Metabolism 2023, 5, 445. 10.1038/s42255-023-00756-4 PMC10202034 36959514

[17] K. Blennow , D. L. Brody , P. M. Kochanek , H. Levin , A. McKee , G. M. Ribbers , K. Yaffe , H. Zetterberg , Nat. Rev. Dis. Primers 2016, 2, 16084. 27853132 10.1038/nrdp.2016.84

[18] D. J. Sharp , G. Scott , R. Leech , Nat. Rev. Neurol. 2014, 10, 156. 24514870 10.1038/nrneurol.2014.15

[19] D. W. Simon , M. J. McGeachy , H. Bayır , R. S. Clark , D. J. Loane , P. M. Kochanek , Nat. Rev. Neurol. 2017, 13, 171. 28186177 10.1038/nrneurol.2017.13 PMC5675525

[20] I. Cernak , L. J. Noble‐Haeusslein , Journal of Cerebral Blood Flow & Metabolism 2010, 30, 255. 19809467 10.1038/jcbfm.2009.203 PMC2855235

[21] C. S. Hill , M. P. Coleman , D. K. Menon , Trends Neurosci. 2016, 39, 311. 27040729 10.1016/j.tins.2016.03.002 PMC5405046

[22] D. G. Siedler , M. I. Chuah , M. T. Kirkcaldie , J. C. Vickers , A. E. King , Frontiers in Cellular Neuroscience 2014, 8, 429. 25565963 10.3389/fncel.2014.00429 PMC4269130

[23] C. M. Luetkemeyer , C. P. Neu , S. Calve , Acta Biomater. 2023, 168, 252. 37433358 10.1016/j.actbio.2023.07.002 PMC10530537

[24] T. Wu , J. A. Rifkin , A. Rayfield , M. B. Panzer , D. F. Meaney , Neuroimage 2022, 251, 119002. 35176490 10.1016/j.neuroimage.2022.119002

[25] A. Fesharaki‐Zadeh , D. Datta , Frontiers in Cellular Neuroscience 2024, 18, 1371213. 38682091 10.3389/fncel.2024.1371213 PMC11045909

[26] V. E. Johnson , W. Stewart , D. H. Smith , Experimental Neurology 2013, 246, 35. 22285252 10.1016/j.expneurol.2012.01.013 PMC3979341

[27] R. J. Cloots , J. Van Dommelen , S. Kleiven , M. Geers , Biomech. Model. Mechanobiol 2013, 12, 137. 22434184 10.1007/s10237-012-0387-6

[28] A. Omelchenko , N. K. Singh , B. L. Firestein , Curr. Opin. Biomed. Eng. 2020, 14, 34. 32671312 10.1016/j.cobme.2020.05.002 PMC7363028

[29] M. E. Hanna , B. J. Pfister , Curr. Opin. Biomed. Eng. 2023, 25, 100430.

[30] K. Pilipović , A. Harej Hrkać , N. Kučić , J. Mršić‐Pelčić , Biomedicines 2022, 11, 94. 36672601 10.3390/biomedicines11010094 PMC9855387

[31] I. Surgucheva , S. He , M. C. Rich , R. Sharma , N. N. Ninkina , P. F. Stahel , A. Surguchov , Mol. Cell. Neurosci. 2014, 63, 114. 25447944 10.1016/j.mcn.2014.10.005

[32] C. Sticozzi , G. Belmonte , A. Meini , P. Carbotti , G. Grasso , M. Palmi , Neuroscience 2013, 252, 367. 23928073 10.1016/j.neuroscience.2013.07.061

[33] A. Marchini , A. Raspa , R. Pugliese , M. A. El Malek , V. Pastori , M. Lecchi , A. L. Vescovi , F. Gelain , Proc. Natl. Acad. Sci. USA 2019, 116, 7483. 30923117 10.1073/pnas.1818392116 PMC6462084

[34] D. Joung , V. Truong , C. C. Neitzke , S. Z. Guo , P. J. Walsh , J. R. Monat , F. Meng , S. H. Park , J. R. Dutton , A. M. Parr , Adv. Funct. Mater. 2018, 28, 1801850. 32595422 10.1002/adfm.201801850 PMC7319181

[35] C. Loussert‐Fonta , L. Stoppini , Y. Neuenschwander , O. Righini , D. Prim , C. Schmidt , M. O. Heuschkel , L. Gomez Baisac , M. Jovic , M. E. Pfeifer , Frontiers in Neuroscience 2023, 17, 1189615. 37397462 10.3389/fnins.2023.1189615 PMC10308006

[36] S. Mobini , Y. H. Song , M. W. McCrary , C. E. Schmidt , Biomaterials 2019, 198, 146. 29880219 10.1016/j.biomaterials.2018.05.012 PMC6957334

[37] R. O. Rodrigues , S.‐R. Shin , M. Bañobre‐López , J. Nanobiotechnol. 2024, 22, 573. 10.1186/s12951-024-02720-0 PMC11409741 39294645

[38] L. Cerutti , M. Brofiga , J. Neurosci. Methods 2024, 405, 110105. 38460796 10.1016/j.jneumeth.2024.110105

[39] S. Nandi , S. Ghosh , S. Garg , S. Ghosh , ACS Chem. Neurosci. 2024, 15, 3828. 39436813 10.1021/acschemneuro.4c00388

[40] C. MacKerron , G. Robertson , M. Zagnoni , T. J. Bushell , Sci. Rep. 2017, 7, 15692. 29146949 10.1038/s41598-017-15950-0 PMC5691080

[41] F. Alghannam , M. Alayed , S. Alfihed , M. A. Sakr , D. Almutairi , N. Alshamrani , N. Al Fayez , Biosensors 2025, 15, 76. 39996978 10.3390/bios15020076 PMC11852457

[42] A. Zare , B. Babamiri , M. Hassani , M. Khalghollah , M. Mohammadi , S. H. Javanmard , A. Sanati Nezhad , Biosens. Bioelectron. 2025, 286, 117599. 40446616 10.1016/j.bios.2025.117599

[43] S. Khetani , K. W. Yong , V. Ozhukil Kollath , E. Eastick , M. Azarmanesh , K. Karan , A. Sen , A. Sanati‐Nezhad , ACS Appl. Mater. Interfaces 2020, 12, 6910. 31971367 10.1021/acsami.9b20826

[44] S. Khetani , K. W. Yong , K. Guan , A. Singh , A. Phani , V. O. Kollath , S. Kim , K. Karan , A. Sen , A. Sanati‐Nezhad , Appl. Mater. Today 2020, 20, 100721.

[45] H. Lee , S. M. Dellatore , W. M. Miller , P. B. Messersmith , Science 2007, 318, 426. 17947576 10.1126/science.1147241 PMC2601629

[46] H. Tolabi , N. Bakhtiary , S. Sayadi , M. Tamaddon , F. Ghorbani , A. R. Boccaccini , C. Liu , Frontiers in Bioengineering and Biotechnology 2022, 10, 1008360. 36466324 10.3389/fbioe.2022.1008360 PMC9715616

[47] D. F. Meaney , D. H. Smith , Clin. Sports Med. 2011, 30, 19. 21074079 10.1016/j.csm.2010.08.009 PMC3979340

[48] I. Mavroudis , D. Kazis , R. Chowdhury , F. Petridis , V. Costa , I.‐M. Balmus , A. Ciobica , A.‐C. Luca , I. Radu , R. P. Dobrin , Diagnostics 2022, 12, 740. 35328293 10.3390/diagnostics12030740 PMC8947595

[49] S. Rowson , S. M. Duma , Ann. Biomed. Eng. 2013, 41, 873. 23299827 10.1007/s10439-012-0731-0 PMC3624001

[50] D. K. Cullen , C. M. Simon , M. C. LaPlaca , Brain Res. 2007, 1158, 103. 17555726 10.1016/j.brainres.2007.04.070 PMC3179863

[51] M. E. Domínguez‐Romero , P. G. Slater , Int. J. Mol. Sci. 2021, 22, 8344. 34361110 10.3390/ijms22158344 PMC8347220

[52] A. C. Bain , D. F. Meaney , J. Biomech. Eng. 2000, 122, 615. 11192383 10.1115/1.1324667

[53] C. Giordano , S. Kleiven , Evaluation of axonal strain as a predictor for mild traumatic brain injuries using finite element modeling, SAE Technical Paper, San Diego, California 2014. 10.4271/2014-22-0002 26192949

[54] S. Rowson , G. Brolinson , M. Goforth , D. Dietter , S. Duma , J. Biomech. Eng. 2009, 131, 061016. 19449970 10.1115/1.3130454

[55] M. Prins , T. Greco , D. Alexander , C. C. Giza , Dis. Models Mech. 2013, 6, 1307. 10.1242/dmm.011585 PMC3820255 24046353

[56] S. P. Broglio , R. Williams , A. Rettmann , B. Moore , J. T. Eckner , S. Meehan , Clinical journal of sport medicine 2018, 28, 130. 28727640 10.1097/JSM.0000000000000420 PMC5767537

[57] B. S. Elkin , J. M. Elliott , G. P. Siegmund , Journal of Orthopaedic & Sports Physical Therapy 2016, 46, 874. 27690834 10.2519/jospt.2016.7049

[58] S. H. North , L. C. Shriver‐Lake , C. R. Taitt , F. S. Ligler , Annu. Rev. Anal. Chem. 2012, 5, 35. 10.1146/annurev-anchem-062011-143105 22462400

[59] H. Zetterberg , K. Blennow , Nat. Rev. Neurol. 2016, 12, 563. 27632903 10.1038/nrneurol.2016.127

[60] J. Gill , M. Mustapic , R. Diaz‐Arrastia , R. Lange , S. Gulyani , T. Diehl , V. Motamedi , N. Osier , R. A. Stern , D. Kapogiannis , Brain Injury 2018, 32, 1359. 10.1080/02699052.2018.1471738 PMC6129391 29913077

[61] M. R. Holahan , Frontiers in Cellular Neuroscience 2017, 11, 266. 28912688 10.3389/fncel.2017.00266 PMC5583208

[62] S. Khetani , A. Singh , B. Besler , S. Butterworth , T. Lijnse , K. Loughery , K. Smith , E. Hosseini , R. Narang , K. Karan , Biosens. Bioelectron. 2021, 178, 113033. 33517230 10.1016/j.bios.2021.113033

[63] S. Khetani , V. O. Kollath , E. Eastick , C. Debert , A. Sen , K. Karan , A. Sanati‐Nezhad , Biosens. Bioelectron. 2019, 145, 111715. 31561093 10.1016/j.bios.2019.111715

[64] S. Khetani , A. Singh , B. Besler , S. Butterworth , T. Lijnse , K. Loughery , K. Smith , E. Hosseini , R. Narang , K. Karan , Biosens. Bioelectron. 2021, 178, 113033. 33517230 10.1016/j.bios.2021.113033

[65] F. K. Korley , V. C. Nikolian , A. M. Williams , I. S. Dennahy , M. Weykamp , H. B. Alam , Journal of Neurotrauma 2018, 35, 1185. 29415612 10.1089/neu.2017.5581

[66] G. TA , Ann. Neurol. 1982, 12, 564.7159060

[67] A. Petzold , Journal of the Neurological Sciences 2005, 233, 183. 15896809 10.1016/j.jns.2005.03.015

[68] D. K. Cullen , J. P. Harris , K. D. Browne , J. A. Wolf , J. E. Duda , D. F. Meaney , S. S. Margulies , D. H. Smith , Injury models of the central nervous system: methods and protocols 2016, 1462, 289.

[69] M. E. Shenton , H. M. Hamoda , J. S. Schneiderman , S. Bouix , O. Pasternak , Y. Rathi , M.‐A. Vu , M. P. Purohit , K. Helmer , I. Koerte , Brain Imaging and Behavior 2012, 6, 137. 22438191 10.1007/s11682-012-9156-5 PMC3803157

[70] A. Ahmad , E. J. A. Moore , The Analyst 2012, 137, 5839. 23099427 10.1039/c2an35236b

[71] Z. Guler , A. J. E. P. L. Sarac , eXPRESS Polymer Letters 2015, 10, 96.

[72] R. Salahandish , F. Haghayegh , G. Ayala‐Charca , J. E. Hyun , M. Khalghollah , A. Zare , B. Far , B. M. Berenger , Y. D. Niu , E. Ghafar‐Zadeh , Biosens. Bioelectron. 2022, 203, 114018. 35114466 10.1016/j.bios.2022.114018 PMC8786409

[73] A. Grillo , G. Wittum , A. Tomic , S. Federico , Math. Mech. Solids 2015, 20, 1107.

[74] R. W. Ogden , Non‐linear Elastic Deformations, Dover, New York, USA 1997.

