# Supplementary material for: A Programmable, 3D Neuron‐On‐Chip Platform Integrating Near Real‐Time Biosensing and Multiaxial Loading for Mechanobiological Injury Profiling
Source: Adv Sci (Weinh). 2025 Oct 7;13(2):e10309. doi: 10.1002/advs.202510309 (PMC12786346; doi:10.1002/advs.202510309)
Supplement: Supplementary file 1 — Supporting Information [file ADVS-13-e10309-s001.docx]

**Supporting Information (SI)**

**A Programmable, 3D Neuron-On-Chip Platform Integrating Near Real-Time Biosensing and Multiaxial Loading for Mechanobiological Injury Profiling**

Sultan Khetani ^1,2^, Kar Wey Yong ^1,2,3^, Mawafag F. Alhasadi ^4^, Amir Hamedzadeh ^4^, Leila Shahsavari ^2^, Atefeh Rafiei ^6^, Anupriya Singh ^1,2^, Kalvin Wu ^1,5^, Omakhowa Agbojo ^1,5^, Kunal Karan ^6^, Salvatore Federico ^2,4,5^, Arindom Sen ^3,6^, Amir Sanati-Nezhad ^1,2,4,5,*^

*^1^ BioMEMS and Bioinspired Microfluidic Laboratory, Department of Biomedical Engineering, University of Calgary, Calgary, Alberta T2N 1N4, Canada*

*^2^ Graduate Program in Biomedical Engineering, University of Calgary, Calgary, Alberta T2N 1N4, Canada*

*^3^ Pharmaceutical Production Research Facility, Schulich School of Engineering, University of Calgary, Alberta T2N 1N4, Canada*

*^4^ Department of Mechanical and Manufacturing Engineering, Schulich School of Engineering, University of Calgary, Calgary, Alberta T2N 1N4, Canada*

*^5^ Department of Biomedical Engineering, Schulich School of Engineering, University of Calgary, Calgary, Alberta T2N 1N4, Canada*

*^6^ Department of Chemical and Petroleum Engineering, University of Calgary, Calgary, Alberta T2N 1N4, Canada*


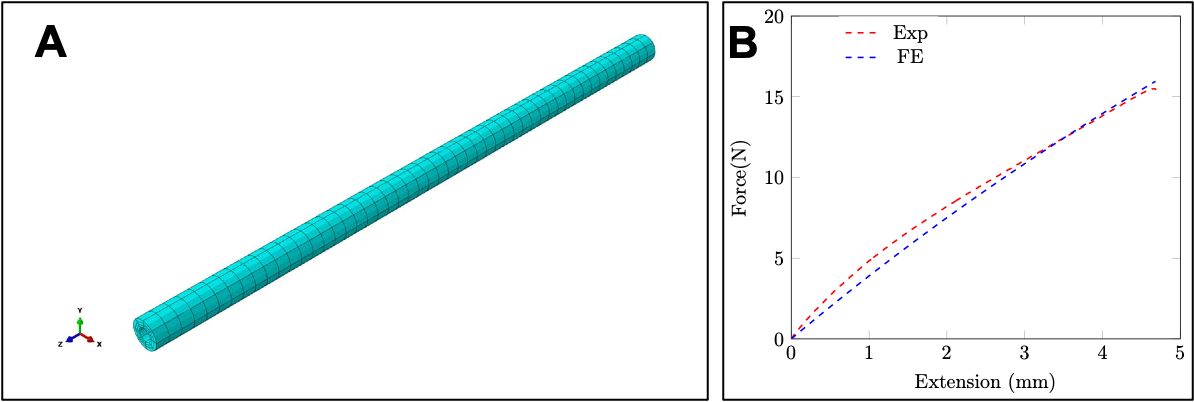


**Figure S1. Finite element (FE) model development and experimental validation.**
(A) Three-dimensional meshing of the cylindrical polydimethylsiloxane (PDMS) tube used in the Neuron-Injury-on-a-Chip (NIOC) platform. The model employs C3D20H hexahedral elements and incorporates a hyperelastic Ogden material model to simulate nonlinear deformation under mechanical loading. The bottom face is fixed, while the top face is displacement-controlled via a kinematic coupling to a reference point. (B) Reaction force versus axial extension curve comparing FE simulation results with experimental data from tension-torsion mechanical tests. The strong agreement validates the accuracy of the Ogden model parameters (μ = 19.14 kPa, α = 1.5, D = 3×10⁻⁸) in reproducing the biomechanical behavior of the PDMS construct under extension.

**Table S1.** Principal Cauchy stress profiles across defined mechanical loading regimes. Summary of finite element modeling results showing the maximum and minimum values of three principal Cauchy stresses ($\sigma_{1}$, $\sigma_{2}$, $\sigma_{3}$) experienced by the neuron-seeded cylindrical PDMS tube under various loading conditions. Cases include uniaxial extension (4–20%), torsion (30°–300°), and combined extension–torsion (4–12% with 30°–90°). The last column reports a scalar measure of stress: the Frobenius norm $\left\| \boldsymbol{\sigma} \right\|=\sqrt{\sigma_{ij} \sigma_{ij}}$ (with Einstein’s convention of sum over the indices $i$ and $j$), which can be equivalently calculated as the Euclidean norm of the vector with components equal to the principal stresses $\sigma_{1}$, $\sigma_{2}$, $\sigma_{3}$). These values provide a quantitative framework to interpret the severity and spatial complexity of mechanical stress distribution applied to the 3D neural construct.

| **Case** | $\sigma_{1}^{max}$ **[Pa]** | $\sigma_{1}^{min}$ **[Pa]** | $\sigma_{2}^{max}$ **[Pa]** | $\sigma_{2}^{min}$ **[Pa]** | $\sigma_{3}^{max}$ **[Pa]** | $\sigma_{3}^{min}$ **[Pa]** | $\left\Vert\boldsymbol{\sigma} \right\Vert$ **[Pa]** |
| --- | --- | --- | --- | --- | --- | --- | --- |
| **Tension**  **4%** | 2.52E+05 | 2.23E+05 | 6.52E+04 | -2.13E+01 | 1.53E+04 | -1.14E+04 | 2.61E+05 |
| **Tension**  **8%** | 5.04E+05 | 4.44E+05 | 1.27E+05 | -2.79E+01 | 3.09E+04 | -2.46E+04 | 5.21E+05 |
| **Tension 12%** | 7.58E+05 | 6.61E+05 | 1.85E+05 | -6.63E+01 | 4.69E+04 | -3.95E+04 | 7.82E+05 |
| **Tension 16%** | 1.01E+06 | 8.76E+05 | 2.41E+05 | -1.42E+02 | 6.30E+04 | -5.59E+04 | 1.04E+06 |
| **Torsion 20%** | 1.27E+06 | 1.09E+06 | 2.93E+05 | -1.93E+02 | 7.96E+04 | -7.38E+04 | 1.30E+06 |
| **Torsion**  **30°** | 2.03E+04 | 1.23E+04 | -9.33E+00 | -3.21E+01 | -1.26E+04 | -2.03E+04 | 2.87E+04 |
| **Torsion**  **60°** | 4.07E+04 | 2.44E+04 | -3.73E+01 | -1.28E+02 | -2.53E+04 | -4.06E+04 | 5.75E+04 |
| **Torsion**  **90°** | 6.11E+04 | 3.63E+04 | -8.39E+01 | -2.89E+02 | -3.83E+04 | -6.09E+04 | 8.63E+04 |
| **Torsion**  **150°** | 1.02E+05 | 5.92E+04 | -2.33E+02 | -8.03E+02 | -6.47E+04 | -1.01E+05 | 1.44E+05 |
| **Torsion 300°** | 2.05E+05 | 1.12E+05 | -9.28E+02 | -3.21E+03 | -1.34E+05 | -2.02E+05 | 2.88E+05 |
| **Tension + Torsion:**  **4% + 30°** | 2.54E+05 | 2.24E+05 | 6.42E+04 | -6.87E+02 | 1.53E+04 | -1.14E+04 | 2.62E+05 |
| **Tension + Torsion:**  **4% + 60°** | 2.60E+05 | 2.27E+05 | 6.11E+04 | -3.15E+03 | 1.52E+04 | -1.15E+04 | 2.67E+05 |
| **Tension + Torsion:**  **4% + 90°** | 2.69E+05 | 2.31E+05 | 5.61E+04 | -8.91E+03 | 1.50E+04 | -1.49E+04 | 2.75E+05 |
| **Tension + Torsion:**  **8% + 30°** | 5.05E+05 | 4.44E+05 | 1.26E+05 | -5.91E+02 | 3.09E+04 | -2.46E+04 | 5.22E+05 |
| **Tension + Torsion:**  **8% + 60°** | 5.09E+05 | 4.45E+05 | 1.25E+05 | -1.55E+03 | 3.08E+04 | -2.46E+04 | 5.25E+05 |
| **Tension + Torsion:**  **8% + 90°** | 5.14E+05 | 4.47E+05 | 1.22E+05 | -3.50E+03 | 3.06E+04 | -2.47E+04 | 5.29E+05 |
| **Tension + Torsion: 12% + 30°** | 7.59E+05 | 6.62E+05 | 1.85E+05 | -3.40E+02 | 4.68E+04 | -3.95E+04 | 7.82E+05 |
| **Tension + Torsion: 12% + 60°** | 7.61E+05 | 6.62E+05 | 1.84E+05 | -1.18E+03 | 4.67E+04 | -3.95E+04 | 7.84E+05 |
| **Tension + Torsion: 12% + 90°** | 7.64E+05 | 6.63E+05 | 1.82E+05 | -2.89E+03 | 4.66E+04 | -3.95E+04 | 7.87E+05 |

**
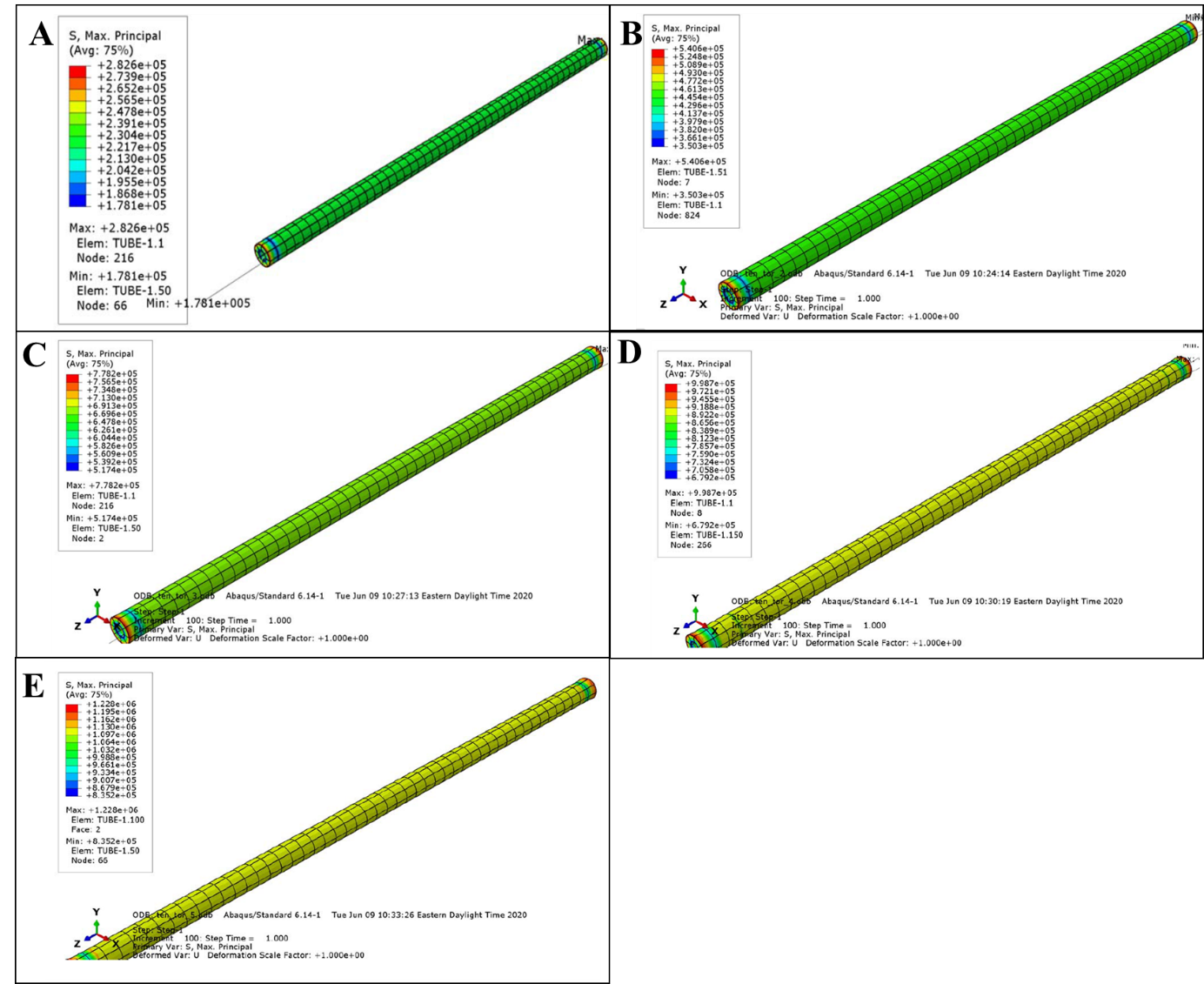
**

**Figure S2**. Finite element simulation of uniaxial extension applied to the NIOC platform. Representative contour plots show the distribution of first principal stress across the neuron-laden cylindrical PDMS construct under increasing tensile strain: (A) 4%, (B) 8%, (C) 12%, (D) 16%, and (E) 20%.

**
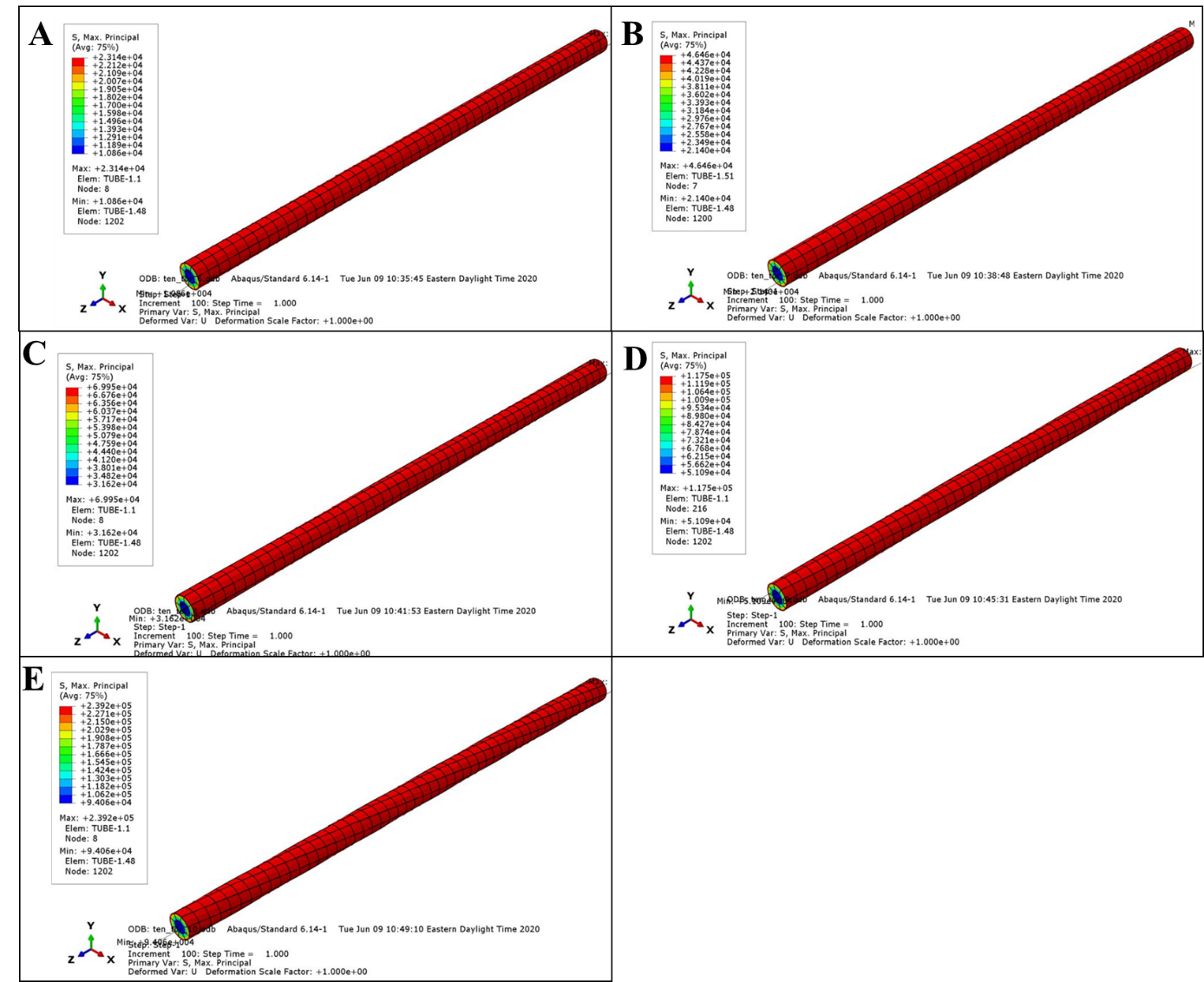
**

**Figure S3. FE simulation of torsional loading applied to the NIOC platform.**
Contour plots illustrate the distribution of first principal stress in the cylindrical PDMS structure subjected to rotational deformation of (A) 30°, (B) 60°, (C) 90°, (D) 150°, and (E) 300°.

**
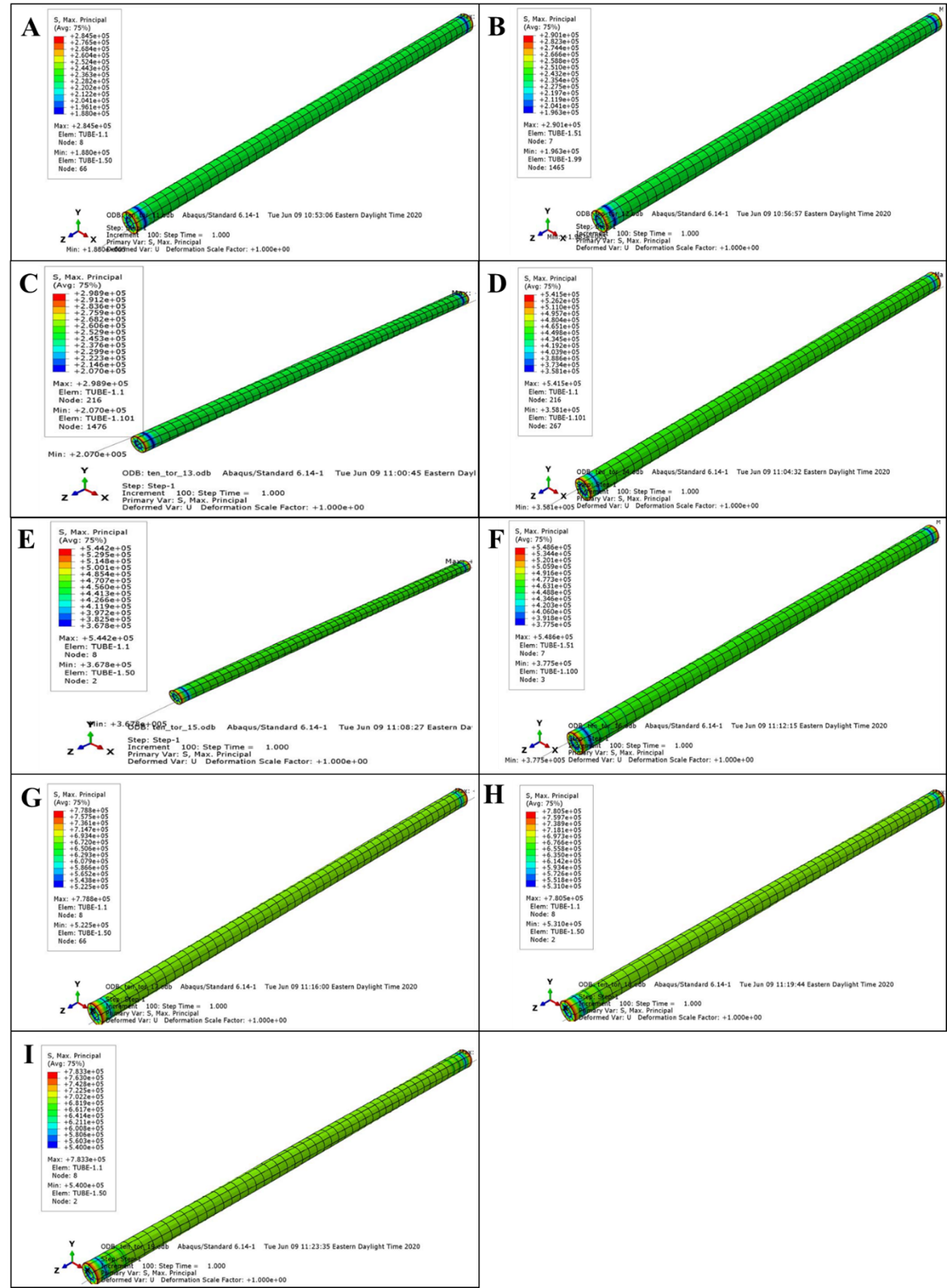
**

**Figure S4**. Finite element simulation results showing first principal stress distributions in the cylindrical microfluidic construct under combined extension and torsion loading conditions: (A) 4% extension + 30° torsion, (B) 4% extension + 60° torsion, (C) 4% extension + 90° torsion, (D) 8% extension + 30° torsion, (E) 8% extension + 60° torsion, (F) 8% extension + 90° torsion, (G) 12% extension + 30° torsion, (H) 12% extension + 60° torsion, and (I) 12% extension + 90° torsion.

**SI-II: Scanning Electron Micrograph (SEM) and Energy Dispersive X-ray Spectroscopy (EDS)**

SEM imaging revealed no significant changes in electrode surface topography during the initial modification steps, including after self-assembled monolayer (SAM) formation and 1-ethyl-3-(3-dimethylaminopropyl)carbodiimide- N-hydroxysuccinimide (EDC–NHS) activation. However, energy dispersive X-ray spectroscopy (EDS) provided molecular-level evidence of surface modification. Increases in nitrogen and carbon signals following EDC–NHS treatment confirmed successful introduction of amine-reactive groups originating from the SAM layer (Figure S5A). Upon antibody conjugation, SEM clearly showed the appearance of discrete globular structures on the gold surface, indicating successful immobilization of antibody molecules enabled by the functionalized chemistry (Figure S5D). These morphological and compositional signatures validated each stage of biosensor fabrication.

**
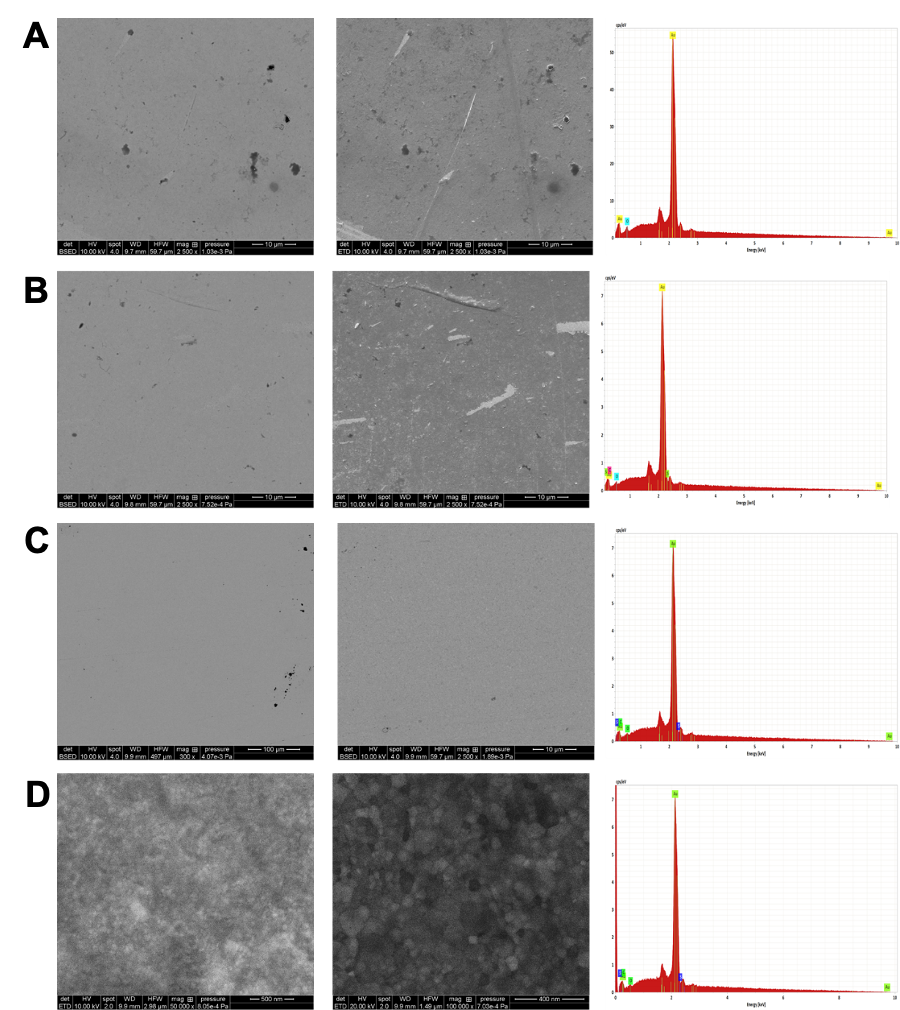
**

**Figure S5**. Scanning electron microscopy (SEM) and energy-dispersive X-ray spectroscopy (EDS) characterization of the biosensor surface at various stages of functionalization. Each row corresponds to a modification stage, with SEM images captured at two magnifications (left and middle columns) and corresponding EDS spectra (right column). From top to bottom: (A) bare gold electrode, (B) self-assembled monolayer (SAM)-modified surface, (C) EDC–NHS activation, and (D) antibody conjugation. The EDS spectra confirm molecular composition changes, notably increased nitrogen and carbon content after EDC–NHS activation, indicating successful amine coupling chemistry. SEM imaging after antibody immobilization reveals the appearance of globular surface structures, consistent with antibody presence.

**SI-III: Diffusion kinetics of the immuno-biosensors**

To evaluate the diffusion behavior of the SAM-modified electrodes, cyclic voltammetry (CV) was performed using a commercial potentiostat in the presence of 4 mM redox couple in phosphate-buffered saline. Scan rates ranging from 10 to 100 mV s⁻¹ were applied to assess redox current response. As shown in Figure S6A, both anodic (Ipa) and cathodic (Ipc) peak currents increased proportionally with scan rate, while peak separation widened at higher scan rates, consistent with diffusion-limited electron transfer. Quantitative analysis revealed a linear relationship between peak current and the square root of the scan rate (v^1/2^), with regression equations $Ipa=0.0034 v^{1/2}-0.0057$ (R² = 0.98) and Ipc = – $Ipc=- 0.008 v^{\frac{1}{2}}+ 0.0053$ (R² = 0.98), confirming diffusion-controlled behavior at the electrode interface (Figure S6B–C). These findings validate the electrode’s performance for biosensing applications governed by analyte diffusion kinetics [1].

**
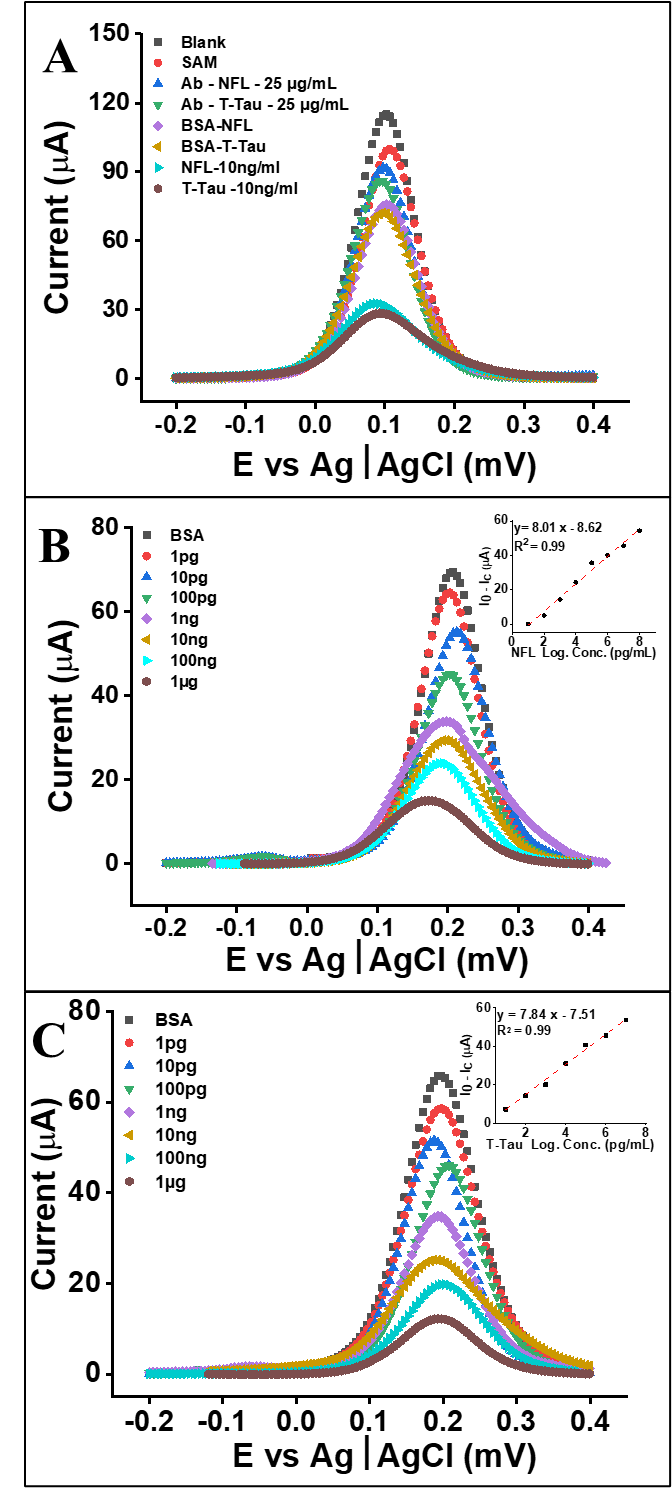
**

**Figure S6**. Electrochemical characterization of diffusion kinetics at the SAM-modified gold electrode surface. (A) Differential pulse voltammetry (DPV) scans recorded at increasing scan rates (10–100 mV s⁻¹) in the presence of 4 mM redox probe. (B) Linear correlation between the anodic peak current (I_pa) and the square root of the scan rate (v^1/2^), indicating diffusion-controlled behavior. (C) Corresponding linear fit for cathodic peak current (Ipc) versus v^1/2^. The results confirm that the redox process is governed by analyte diffusion, validating the electrode interface for biosensing applications.

**SI-IV: MALDI-TOF mass spectrometry analysis of PDA stability under mechanical loading**

To verify the chemical resilience of the polydopamine (PDA) coating used in the NIOC platform, we performed matrix-assisted laser desorption/ionization time-of-flight mass spectrometry (MALDI-TOF MS) on conditioned culture media collected after 120 min of mechanical loading. PDA serves as a critical interfacial layer promoting uniform cell adhesion and surface biocompatibility; however, its behavior under prolonged mechanical stress had not been previously validated in microfluidic systems.

To assess possible leaching or degradation, we compared post-loading spectra against controls including individual reference spectra for the MALDI matrix, Tris buffer, dopamine (DA) monomer, PDA oligomers, and trypsin (used during prior electrode cleaning steps). The characteristic mass peaks for these species are summarized in Table S2. Importantly, no PDA-related peaks were detected in the media samples from mechanically stimulated chips, suggesting no detectable delamination or release of PDA fragments into the surrounding medium. This confirms that the PDA layer remains chemically intact and covalently bound under all applied loading conditions, including extension, torsion, and combined stresses. The findings validate the mechanical and chemical stability of the PDA-modified PDMS microtubes, supporting their use in sustained biomechanical experimentation and longitudinal biosensing applications. The robustness of the PDA coating is critical for ensuring consistent neuronal adhesion and eliminating spurious signal artifacts due to coating degradation or leachate interference during real-time biosensor operation.

**Table S2**. Reference mass spectrometry peaks for MALDI matrix, buffer components, monomers, polymers, and media additives.

| **Compound** | **Peaks** | **Reference** |
| --- | --- | --- |
| **MALDI matrix** | HCCA+H = 190.05, HCCA+Na = 212.03,  NCCA+K = 238.005, 2HCCA+H = 379.09,  2HCCA+Na = 401.07, 2HCCA+K = 417.04,  Acetalidine + H = 136.07, Acetalidine + Na =158 05,  Acetalidine + K = 174.03 | **[2]** |
| **Tris buffer** | 122 | **[3]** |
| **Dopamine (DA)** | 154.4 |  |
| **Polydopamine (PDA)** | 402.2 |  |
| **Trypsin** | 805.9, 907.1, 1112.3, 1154.3,  1434.7, 2164.3, and 2274.6 | **[4]** |

**
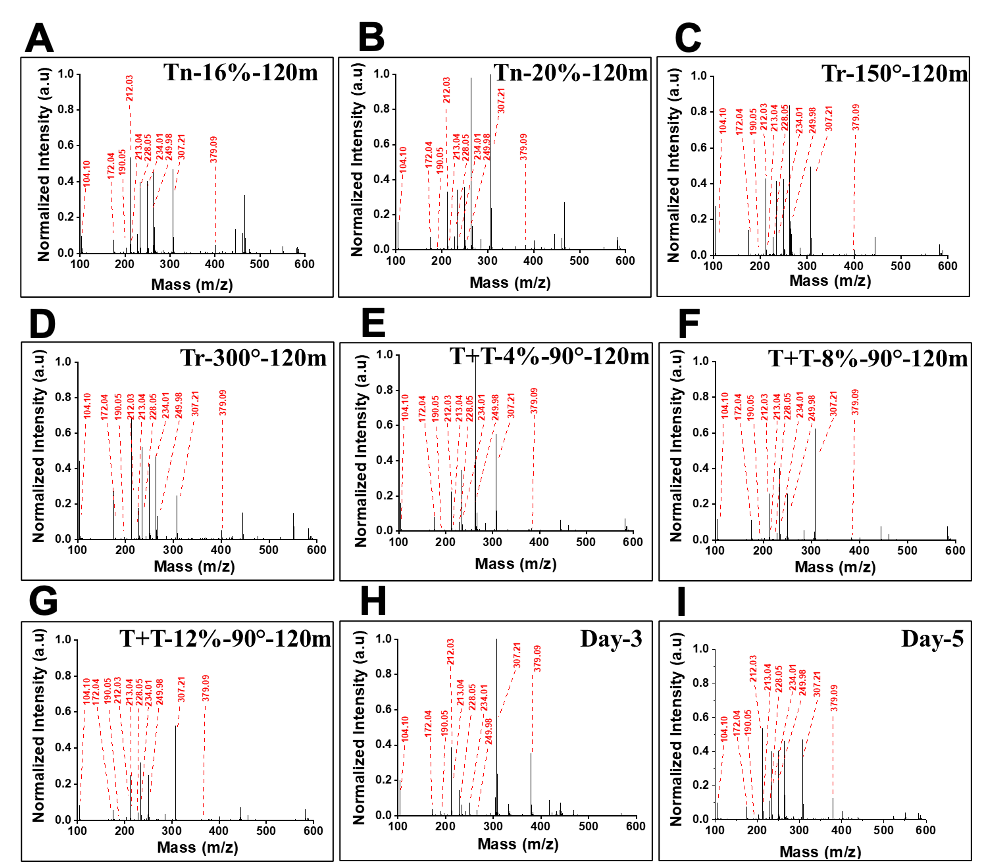
**

**Figure S7**. MALDI-ToF-MS spectra of conditioned culture media collected 120 minutes after application of mechanical loading to the 3D NIOC platform. Panels represent media samples following (A,B) uniaxial extension at 16% and 20%, (C,D) torsional loading at 150° and 300°, and (E-G) combined extension–torsion at 4%–90°, 8%–90°, and 12%–90°, respectively. Control spectra from CAD neuron-seeded microtubes cultured without mechanical loading are shown for (H) 3 days and (I) 5 days. No distinct peaks attributable to polydopamine (PDA) degradation or electrode leaching were observed, supporting the chemical integrity of the platform under dynamic loading conditions.

**Table S3**. Summary of operational parameters for electrochemical immuno-biosensors developed for the detection of tau isoforms (C-Tau, T-Tau) and neurofilament light chain (NFL) proteins across various biofluids.

| **Method** | **Limit of detection** | **Analytical range** | **Sample matrix** | **Ref.** |
| --- | --- | --- | --- | --- |
| **Tau (C-Tau or T-Tau)** | | | | |
| Optical sensors | 1.91 pg mL^-1^ | 15.6 pg/ml  -  2000 pg/ | PBS, CSF | [5] |
| Electrochemical biosensor | 0.03 pM, | 10^-14^ - 10 ^-5^ M | PBS, serum | [6] |
| Immuno-Infrared sensor | - | - |  | [7] |
| Reduced graphene oxide biosensor | 75 fm | 0.08 pM - 80 pM | PBS, serum | [8] |
| Electrochemical biosensor (DPV) | Benchtop potentiostat:  0.32 pg/mL (in PBS)  μDrop:  0.14 pg/mL (in PBS)  0.10 pg/mL (in Serum) | Benchtop potentiostat: 1 pg/mL – 1 μg/mL  μDrop:  10 pg/mL – 100 ng/mL | PBS, serum | This work |
| **NFL** | | | | |
| Electrochemical biosensor | 5.21 ng/L | 1 μg/L to 50 μg/L | CSF | [9] |
| SIMOA-Single plex assay | 0.104 pg/mL | - | plasma | [10] |
| Electrochemical biosensor (DPV) | Benchtop potentiostat: 0.14 pg/mL (in PBS)  μDrop:  0.13 pg/mL (in PBS)  0.11 pg/mL (in Serum) | Benchtop potentiostat:  1 pg/mL – 1 μg/mL  μDrop:  10 pg/mL – 100 ng/mL | PBS, DMEM cell media | This work |

**SI-V: Differential expression of NFL and T-Tau after mechanical damage of the 3D-neuron tissue inside the NIOC**

To assess the biomarker specificity of the NIOC platform under diverse mechanical stress conditions, we performed a comparative quantification of T-Tau and NFL release across varying intensities of extension, torsion, and combined mechanical loads (Figure S7). Results indicate a distinct load-dependent expression pattern for both biomarkers. T-Tau levels demonstrated a sharp increase under higher extension (>12%) and torsional (>150°) stress, with synergistic elevation observed in combined loading regimes (e.g., 12% + 90°), consistent with cytoskeletal destabilization and microtubule damage. NFL, a marker of axonal disruption, showed a more gradual increase but surpassed T-Tau at extreme load conditions, aligning with axon-specific damage. The multiplexed biosensing data validate the NIOC system’s capacity to discriminate between different injury profiles and detect distinct mechanosensitive biomarkers with high temporal resolution. These trends reinforce the potential for T-Tau and NFL to serve as complementary indicators of force-specific neuronal damage in translational neurotrauma diagnostics.

**
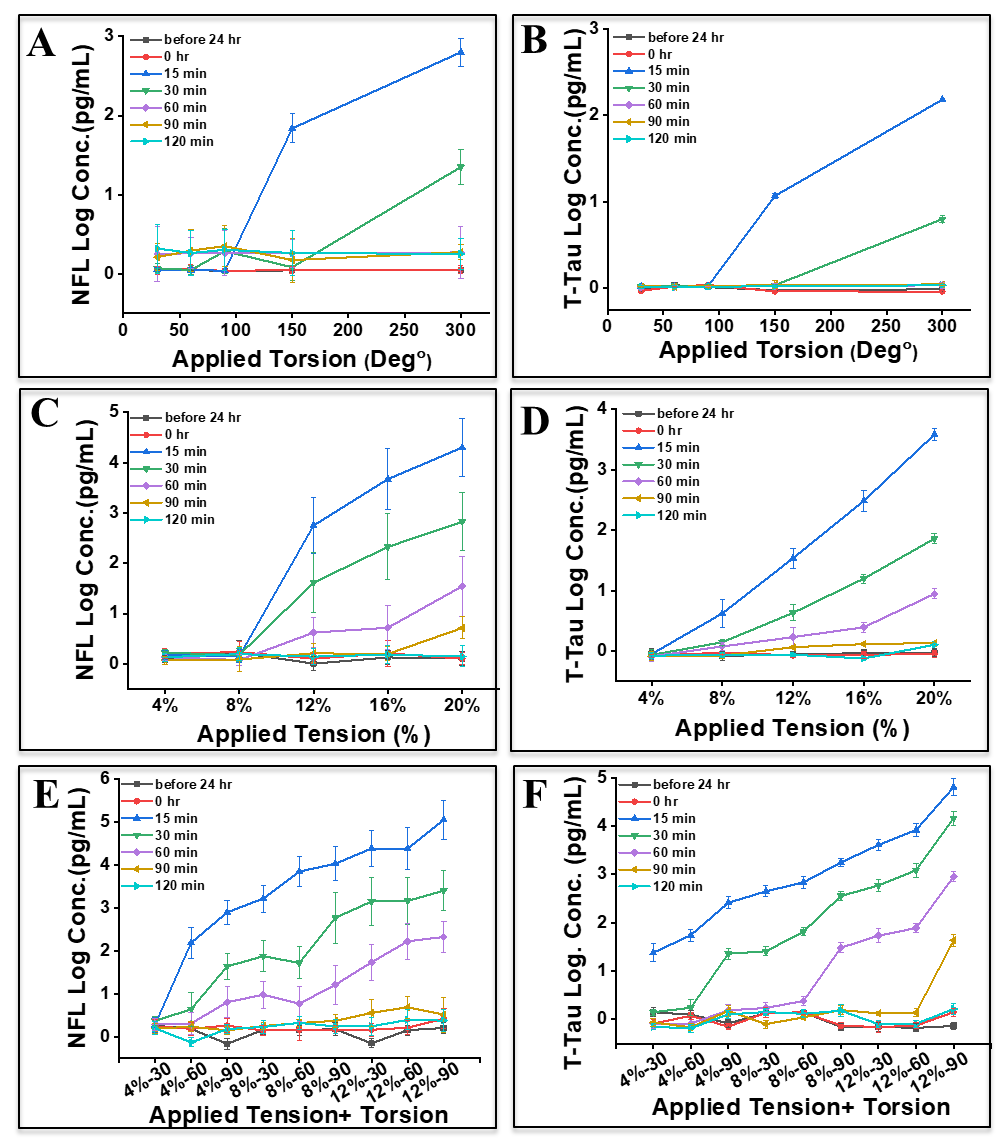
**

**Figure S8.** Quantification of T-Tau (left panels) and NFL (right panels) protein concentrations measured in conditioned medium after subjecting the 3D neuronal construct to (A, B) uniaxial extension (4–20%), (C, D) torsion (30°–300°), and (E, F) combined extension–torsion loads (4–12% + 30°–90°). Data were acquired using multiplexed electrochemical biosensors and are presented as mean ± SEM from three independent experiments.


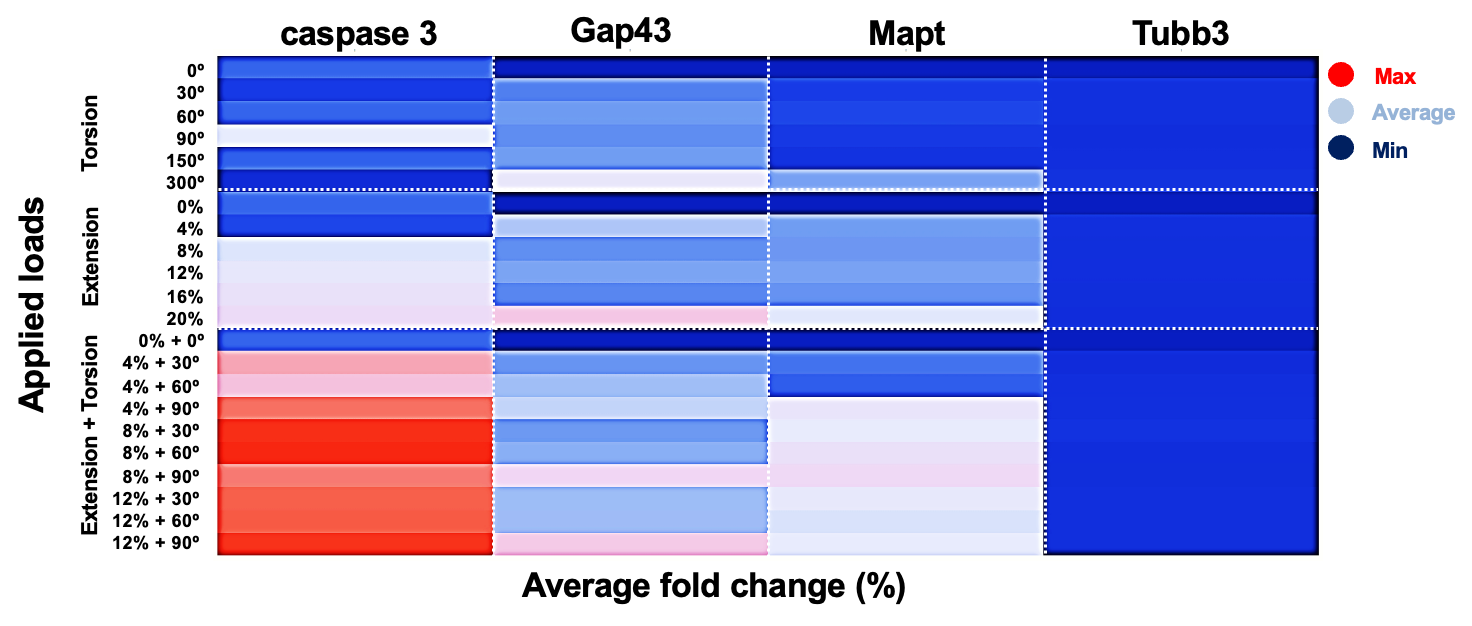


**Figure S9. Heat map representation of gene expression following mechanical loading.** Relative mRNA expression levels of Mapt, Gap-43, and Tubb3 measured by qRT-PCR 15 minutes after the application of mechanical loads (uniaxial extension, torsion, and combined extension–torsion) to CAD neurons cultured within the NIOC platform. Fold changes are shown relative to static controls. The heat map highlights load-specific transcriptional responses, with Mapt and Gap-43 showing pronounced upregulation under high-strain or combined loading, while Tubb3 remains largely unchanged, indicating preservation of general neuronal identity.

To contextualize these biosensor-measured concentrations, **Supplementary Table 4** compiles clinically reported T-Tau and NFL levels in serum/plasma and CSF across TBI severities, together with our NIOC measurements. This comparison demonstrates that the peak values observed in NIOC fall within the ranges measured in acute blood samples from TBI patients, while remaining below typical CSF levels.

**Table S4**. Clinically reported concentration ranges for T-Tau and NFL in serum/plasma and CSF following traumatic brain injury.

| **Reference** | **Biofluid** | **Injury Severity / Context** | **T-Tau (pg·mL⁻¹)** | **NFL (pg·mL⁻¹)** | **Notes** |
| --- | --- | --- | --- | --- | --- |
| Zetterberg & Blennow [11] | Serum/Plasma | Acute mTBI–Moderate TBI | ~5–50 | ~20–150+ | Benchmark review of TBI biomarkers |
| Korley et al., [12] | Plasma | ED mild–moderate TBI | 6–40 | 25–80 | Acute-phase plasma measurements |
| Gaetani et al., [13] | CSF | Moderate–Severe TBI | 500–1500 | 1000–5000 | Confirms high CSF values |
| Shahim et al., [14] | Serum/CSF | Mild–Moderate TBI; longitudinal | 10-20 | hundreds–thousands | Serum-CSF correlation and kinetics |
| Koerbel et al., [15] | Serum/CSF | Acute TBI | Serum NFL ≈10.6 (IQR ≈9.4) | CSF NFL ≈1453 (IQR ≈2456) | Demonstrates ~100× CSF/serum gap |
| Kaaber et al., [16] | Serum | Acute TBI, outcome association | low–mid pg·mL⁻¹ | low–mid pg·mL⁻¹ | Acute serum levels predict outcomes |

Abbreviations: CSF – cerebrospinal fluid; NFL – neurofilament light chain; T-Tau – total tau protein; IQR – interquartile range; ED – emergency department; mTBI – mild traumatic brain injury.

**References**

[1] X. Du, Z. Zhang, X. Zheng, H. Zhang, D. Dong, Z. Zhang, M. Liu, J. J. N. C. Zhou, **2020**, *11* (1), 1.

[2] S. Khetani, K. W. Yong, K. Guan, A. Singh, A. Phani, V. O. Kollath, S. Kim, K. Karan, A. Sen, A. Sanati-Nezhad, *Applied Materials Today* **2020**, *20*, 100721.

[3] Y. Yang, P. Qi, Y. Ding, M. F. Maitz, Z. Yang, Q. Tu, K. Xiong, Y. Leng, N. Huang, *Journal of Materials Chemistry B* **2015**, *3* (1), 72.

[4] S. Khetani, K. W. Yong, V. Ozhukil Kollath, E. Eastick, M. Azarmanesh, K. Karan, A. Sen, A. Sanati-Nezhad, *ACS Applied Materials & Interfaces* **2020**, *12* (6), 6910.

[5] C. Song, P. Deng, L. J. N. N. Que, Biology, Medicine, **2018**, *14* (6), 1845.

[6] S. X. Wang, D. Acha, A. J. Shah, F. Hills, I. Roitt, A. Demosthenous, R. H. J. B. Bayford, Bioelectronics, **2017**, *92*, 482.

[7] B. Budde, J. Schartner, L. Tönges, C. Kötting, A. Nabers, K. J. A. s. Gerwert, **2019**, *4* (7), 1851.

[8] M. Ye, M. Jiang, J. Cheng, X. Li, Z. Liu, W. Zhang, S. M. Mugo, N. Jaffrezic-Renault, Z. J. S. Guo, A. B. Chemical, **2020**, 127692.

[9] E. Özgür, H. U. Uyanık, S. Şenel, L. J. M. S. Uzun, E. B, **2020**, *256*, 114545.

[10] F. K. Korley, J. K. Yue, D. H. Wilson, K. Hrusovsky, R. Diaz-Arrastia, A. R. Ferguson, E. L. Yuh, P. Mukherjee, K. K. Wang, A. B. J. J. o. N. Valadka, **2019**, *36* (1), 182.

[11] H. Zetterberg, K. Blennow, *Nature Reviews Neurology* **2016**, *12* (10), 563.

[12] F. K. Korley, J. K. Yue, D. H. Wilson, K. Hrusovsky, R. Diaz-Arrastia, A. R. Ferguson, E. L. Yuh, P. Mukherjee, K. K. Wang, A. B. Valadka, *Journal of Neurotrauma* **2019**, *36* (1), 182.

[13] L. Gaetani, K. Blennow, P. Calabresi, M. Di Filippo, L. Parnetti, H. Zetterberg, *Journal of Neurology, Neurosurgery & Psychiatry* **2019**, *90* (8), 870.

[14] P. Shahim, Y. Tegner, N. Marklund, K. Blennow, H. Zetterberg, *Neurology* **2018**, *90* (20), e1780.

[15] K. Koerbel, M. Maiworm, M. Schaller-Paule, J. H. Schäfer, J. Jakob, L. Friedauer, F. Steffen, S. Bittner, C. Foerch, Y. Yalachkov, *Multiple Sclerosis and Related Disorders* **2024**, *87*, 105644.

[16] I. A. Kaaber, M. Lesbo, T. O. Wichmann, D. A. Olsen, M. M. Rasmussen, O. Brink, L. C. Borris, C. V. Hviid, *Scientific Reports* **2024**, *14* (1), 14139.
